# Supplementary material for: Planthopper salivary sheath protein LsSP1 contributes to manipulation of rice plant defenses
Source: Nat Commun. 2023 Feb 10;14:737. doi: 10.1038/s41467-023-36403-5 (PMC9911632; doi:10.1038/s41467-023-36403-5)
Supplement: Supplementary file 1 — Supplementary Information [file 41467_2023_36403_MOESM1_ESM.pdf]

Supplementary Information for

Planthopper salivary sheath protein LsSP1 contributes to manipulation of rice plant defenses

Hai-Jian Huang[1,2], Yi-Zhe Wang[1,2], Li-Li Li[1,2], Hai-Bin Lu[1,2], Jia-Bao Lu[1,2], Xin Wang[1,2], Zhuang-Xin Ye[1,2], Ze-Long Zhang[1,2], Yu-Juan He[1,2], Gang Lu[1,2], Ji-Chong Zhuo[1,2], Qian-Zhuo Mao[1,2], Zong-Tao Sun[1,2], Jian-Ping Chen[1, 2 #], Jun-Min Li[1, 2 #], Chuan-Xi Zhang[1,2 #]

[1] State Key Laboratory for Managing Biotic and Chemical Threats to the Quality and Safety of Agro-products, Institute of Plant Virology, Ningbo University, Ningbo 315211, China

[2] Key Laboratory of Biotechnology in Plant Protection of MARA and Zhejiang Province, Institute of Plant Virology, Ningbo University, Ningbo 315211, China

# Corresponding authors: Jian-Ping Chen, jpchen2001@126.com; Jun-Min Li, lijunmin@nbu.edu.cn; Chuan-Xi Zhang, chxzhang@zju.edu.cn

Supplementary Notes: 1-5

Supplementary Methods: 1

Supplementary Figures: 1-23

Supplementary Tables: 1-4

Supplementary References: 1-9

Supplementary Notes 1-5

Supplementary Note 1. Interactions between LsSP1 and rice papain-like cysteine proteases (PLCPs) revealed by Y2H, GST-pull down, BiFC and LUC assays

Point-to-point Y2H assays revealed that both the immature OsOryzain (iOsOryzain) and mature OsOryzain (mOsOryzain) interacted with LsSP1, while the self-inhibitory prodomain or granulin domain did not, indicating that OsOryzain bound to LsSP1 via the peptidase domain (Supplementary Fig. 7a, b). Besides, the interaction between mOsOryzain and LsSP1 was confirmed using GST-pull down, BiFC, and LUC assays (Supplementary Fig. 7c-f).

Totally 46 canonical PLCPs were identified by searching the *Oryza sativa* genome, which were grouped into 9 subfamilies based on their homology to the previously categorized PLCPs[1] (Supplementary Fig. 8). Since PLCPs shared conserved peptidase domains (Supplementary Fig. 8), this study examined the potential interaction between LsSP1 and PLCPs from other subfamilies. In total, 15 PLCPs from 9 subfamilies were tested. In addition to OsOryzain, another 9 PLCPs were confirmed to interact with LsSP1, including Os04g57440, Os09g27030, Os05g24550, Os04g24600, Os05g01810, Os08g44270, Os09g39060, Os09g38920 and Os04g01710 (Supplementary Fig. 8, Supplementary Fig. 9). There was no direct correlation of PLCP phylogeny with PLCP-LsSP1 interaction. For example, three PLCPs (Os08g44270, Os09g39170, and Os09g39060) from CEP1 subfamily showed different binding abilities to LsSP1. The binding specificity between PLCPs and LsSP1 deserves further investigation. Nonetheless, our experiments suggest that LsSP1 interacts with multiple PLCPs belonging to different subfamilies in Y2H and BiFC assays.

Supplementary Note 2. Induction of OsOryzain by *Laodelphax striatellus* infestation and salicylic acid (SA) treatment using rice plants

The protein levels of OsOryzain in response to SA treatment and *L. striatellus* infestation were determined by Western-blotting assay. At first, the OsOryzain abundance in rice sheath was investigated. Two bands corresponding to iOsOryzain and mOsOryzain were detected (Supplementary Fig. 11b), similar to C14 in tomato. Overall, the abundance of iOsOryzain in rice sheath was higher than that of mOsOryzain. At 12 and 24 h after SA treatment or *L. striatellus* infestation, both iOsOryzain and mOsOryzain levels significantly increased (Supplementary Fig. 11b). Thereafter, the OsOryzain abundance in apoplast was also investigated. The abundance of

mOsOryzain in rice apoplast was higher than that of iOsOryzain. The elevated mOsOryzain level in rice apoplast was detected at 12 h after SA treatment, but not after *L. striatellus* infestation (Supplementary Fig. 11c). At 24 h, both SA treatment and *L. striatellus* infestation induced an increased mOsOryzain level in rice apoplast than that in the control. However, the apoplastic mOsOryzain level under SA treatment was still higher than that under *L. striatellus* infestation (Supplementary Fig. 11c). These results demonstrate that SA treatment and *L. striatellus* infestation induce the expression of mOsOryzain in plant cells, while rice plants infested by *L. striatellus* secrete a lower amount of mOsOryzain into apoplast than that under SA treatment. We presumed that *L. striatellus* potentially reduced the level of mOsOryzain in apoplast.

Supplementary Note 3. LsSP1 restricts OsOryzain to salivary sheath and inhibits the activity of apoplastic proteases

The salivary sheath protein LsSP1 was confirmed to interact with OsOryzain using Y2H, GST-pull down, BiFC, and LUC assays (Supplementary Fig. 7). We wonder whether OsOryzain can bind to salivary sheath. To this end, *L. striatellus* were initially allowed to feed on an artificial diet, and the parafilm attached with salivary sheath was incubated with mOsOryzain-GFP and GFP proteins. As a result, strong green fluorescence signal was detected in salivary sheath incubated with mOsOryzain-GFP (Supplementary Fig. 13a). By contrast, only a weak fluorescence signal was detected in the control (Supplementary Fig. 13a). Subsequently, *L. striatellus* were pre-treated with ds*GFP* and ds*LsSP1*, and the binding capacity of the secreted salivary sheaths was tested with mOsOryzain-GFP. The results revealed that the green fluorescence intensity decreased in salivary sheaths secreted from ds*LsSP1*-treated *L. striatellus* than that secreted from the ds*GFP*-treated ones (Supplementary Fig. 13b), suggesting that LsSP1 is important for binding to OsOryzain *in vitro*. Also, rice plants infested by ds*GFP*- and ds*LsSP1*-treated *L. striatellus* were incubated with the anti-LsSP1 serum conjugated with Alexa Fluor™ 488 (green) and anti-OsOryzain serum conjugated with Alexa Fluor™ 555 (red), respectively. According to the obtained results, the green and red fluorescence signals were overlapped in ds*GFP*-treated *L. striatellus* infested plants, with the strongest OsOryzain signal being detected around the salivary sheath (Supplementary Fig. 13c). Comparatively, the Alexa Fluor™ 555 signal (OsOryzain protein) in ds*LsSP1*-treated *L. striatellus* infested plants did not cluster around the salivary sheath (Supplementary Fig. 13c), suggesting that

OsOryzain fails to bind to salivary sheath in the absence of LsSP1 in rice plants.

The proteolytic activity plays a critical role in activating plant immune responses, and the plant apoplast contains high levels of proteases. In this study, the inhibitory effect of LsSP1 on the proteolytic activity of apoplastic proteases was determined using the protease assay kit. The reconstituted BODIPY-FL casein was used as the substrate, whereas the chemical inhibitor E64 and the proteinase inhibitor cocktail (PI) were applied as the positive controls. Both E64 and PI efficiently inhibited the activities of apoplastic proteases at the concentrations of 100 nM and 500 nM, respectively (Supplementary Fig. 13d). Compared with GST protein treatment, a significant decrease in proteolytic activity was detected after LsSP1-GST protein treatment at a concentration of 500 nM (two-tailed unpaired Student's *t*-test, p = 0.0013; Supplementary Fig. 13d). At a concentration of 100 nM, a slight decrease, but of no statistical significance, was detected after LsSP1-GST protein treatment compared with the control (two-tailed unpaired Student's *t*-test, p = 0.1003; Supplementary Fig. 13d). These results may suggest that LsSP1 is capable of inhibiting the proteolytic activity of apoplastic proteases. Apoplastic proteases are the targets of multiple pathogen-derived inhibitors, and one inhibitor can target multiple proteases[4]. Interestingly, LsSP1 and other inhibitors did not exhibit sequence similarity or were even restricted to specific species. Further analyses are needed to investigate the mechanism of LsSP1 in inhibiting plant proteolytic activity.

Supplementary Note 4. LsSP1 reduces plant defenses triggered by OsOryzain and mucin-like protein (LsMLP) in *Nicotiana benthamiana* plants

OsOryzain with a GFP tag was transiently expressed in *N. benthamiana* via *Agrobacterium* infiltration. As a result, OsOryzain-GFP was mainly located in the endoplasmic reticulum (ER) and vacuoles, but not in the Golgi (Supplementary Fig. 14a), in line with the location pattern of RD21[5]. The ER- and vacuole-associated localization of OsOryzain was mainly correlated with the peptidase domain, whereas the GFP-tagged inhibitory domain showed distinct localization patterns (Supplementary Fig. 14b). Interestingly, the expression of OsOryzain-GFP, iOsOryzain-GFP, and mOsOryzain-GFP induced H_2O_2 accumulation in *N. benthamiana* (Supplementary Fig. 14c). On the contrary, GFP, LsSP1-GFP, and inhibitory domain-GFP expression did not trigger H_2O_2 accumulation (Supplementary Fig. 14c).

To investigate the effect of LsSP1 on the OsOryzain-induced plant defense in *N. benthamiana*, LsSP1 with a mCherry tag (LsSP1-mCherry) was co-expressed with mOsOryzain-GFP. As a result, the localization patterns of mOsOryzain were changed in the presence of LsSP1-mCherry, and both fluorescence signals were colocalized in the cytomembrane (Supplementary Fig. 15a). By contrast, the expression of RFP-mCherry protein did not affect the localization patterns of mOsOryzain-GFP, and two fluorescence signals were not overlapped (Supplementary Fig. 15a). LsSP1-mCherry expression attenuated H_2O_2 accumulation caused by mOsOryzain-GFP expression (Supplementary Fig. 15b), suggesting that LsSP1 may suppress the mOsOryzain-induced H_2O_2 accumulation in *N. benthamiana*. In addition, mOsOryzain-GFP expression elicited upregulation of 4 pathogenesis-related (PR) proteins (Supplementary Fig. 15d). Moreover, the jasmonic acid (JA)-responsive gene *plant defensing 1.2* (*NbPDF1.2*) was suppressed upon mOsOryzain-GFP expression, while the SA-responsive gene *non-expresser of PR genes 1* (*NbNPR1*) was induced (Supplementary Fig. 15d), consistent with the findings that PLCPs activated SA signal[6]. When LsSP1-mCherry was co-expressed with mOsOryzain-GFP, the expression levels of *NbPR1*, *NbPR3*, and *NbPR4* significantly decreased in comparison with those after co-expression of RFP-mCherry with mOsOryzain-GFP (Supplementary Fig. 15d). These results show that LsSP1 reduces the OsOryzain-triggered plant defenses in *N. benthamiana*.

As reported in a previous study, MLP secreted from *N. lugens* is one of the HAMPs that are capable of inducing plant defenses[7]. This study firstly investigated the potential role of LsMLP by transiently expressing LsMLP-GFP in *N. benthamiana*, and found that LsMLP-GFP expression resulted in H_2O_2 accumulation (Supplementary Fig. 14c). Subsequently, the impact of LsSP1 on LsMLP-induced plant defenses was evaluated. To this end, LsSP1-mCherry or RFP-mCherry was co-expressed with LsMLP-GFP in *N. benthamiana*. The attenuated H_2O_2 accumulation was observed after co-expression of LsMLP-GFP with LsSP1-mCherry (Supplementary Fig. 14c). Also, the expression patterns of *NbPR2*, *NbPR3*, *NbPR4*, and *NbNPR1*, which were induced by LsMLP-GFP, were significantly suppressed in the presence of LsSP1-mCherry (Supplementary Fig. 14e). In contrast, the expression pattern of *NbPDF1.2*, which was suppressed by LsMLP-GFP, was recovered in the presence of LsSP1-mCherry (Supplementary Fig. 14e). Based on the above findings, LsSP1 is capable of inhibiting the LsMLP-triggered plant defenses in *N. benthamiana*.

Supplementary Note 5. Effects of dsRNA-treated *L. striatellus* on *koOry* plants

Transgenic Nipponbare rice plants with OsOryzain knockout were constructed (Supplementary Fig.17b, c). The wild type (WT) Nipponbare plant was used as a control. Two independent homozygous lines (*koOry#1* and *koOry#2*) were used, and similar results were obtained. There was no significant resistance change in *koOry* plants when compared with WT plants (two-tailed unpaired Student's *t*-test, p = 0.1708 in comparison group 1, p = 0.1210 in comparison group 2; Supplementary Fig. 19). Also, the treatment of *L. striatellus* with ds*LsSP1* did not affect insect survivorship (log-rank test, p = 0.9813 for *koOry#1*, p = 0.7145 for *koOry#2*; Supplementary Fig. 20) or honeydew excretion (two-tailed unpaired Student's *t*-test, p = 0.4584 in comparison group 1, p = 0.3713 in comparison group 2; Supplementary Fig. 21b, d) when compared with the ds*GFP* treatment. However, the fecundity of ds*LsSP1*-treated *L. striatellus* was lower than the ds*GFP*-treated control, although with no statistical significance (two-tailed unpaired Student's *t*-test, p = 0.7882 in comparison group 1, p = 0.3282 in comparison group 2; Supplementary Fig. 21a, c). EPG was used to monitor the insect feeding behavior on *koOry* plants. Compared with ds*GFP*-treated *L. striatellus*, a slight increase in pathway duration phase was observed in ds*LsSP1*-treated *L. striatellus* (two-tailed unpaired Student's *t*-test, p = 0.0245 in comparison group 1, p = 0.0537 in comparison group 2; Supplementary Fig. 21e, f). These results demonstrate that rice plants knockout of OsOryzain cannot well rescue the feeding defects caused by a deficiency in LsSP1 secretion as that LsSP1 overexpressing plants did.

Transcriptomic analyses were also performed on *koOry#1* plants that were untreated or infested by ds*LsSP1*-treated *L. striatellus*. Totally 1532 DEGs were identified in *koOry#1* plants (Supplementary Data 5), which was less than that of WT plants (3396 DEGs). There were 2389 DEGs specifically identified in WT plants, but not in *koOry#1* plants, and they were potentially correlated with OsOryzain-associated responses. Enrichment analysis revealed that the majority of these genes were involved in plant hormone signal transduction, plant-pathogen interaction, MAPK signal transduction, and environmental adaptation (Supplementary Fig. 22a). Among the 16 SA-related genes that were significantly up-regulated after ds*LsSP1*-treated *L. striatellus* infestation in WT plants, 9 genes were induced to a lower extent in *koOry#1* plants compared with those in WT plants, indicating that OsOryzain knockout may attenuate the *L. striatellus*-induced SA biosynthesis and SA response (Supplementary Fig. 22b).

Supplementary Methods

Protein-protein interaction (PPI) assays

In the Y2H screening assay, the coding sequence of LsSP1 was constructed into the pGBKT7 vector (Clontech, Mountain View, CA, USA) (Supplementary Table 3), whereas the cDNA library of *O. sativa* was constructed into pGADT7 vector (Clontech). Later, the recombinant vectors were co-transfected into the yeast strain Y2H Gold, and the positive clones were selected on quadruple dropout (QDO) solid medium (SD/−adenine/−histidine/−leucine/−tryptophan) (#630428, Takara) for approximately 3 days at 30 °C. After colonies were harvested from the QDO liquid medium, the positive yeast plasmids were extracted using the TIANprep Yeast plasmid DNA kit (#DP112-02, TIANGEN, Beijing, China) and subsequently introduced into *Escherichia coli* DH5α competent cells (TSC-C14, Tsingke), so as to identify the potential interacting genes by Sanger sequencing (YouKang Biotech, Hangzhou, China).

In the Y2H point-to-point verification assay, PLCPs, LsMLP, LsSP1, and OsOryzain of different isoforms were cloned into pGBKT7 or pGADT7 vector, respectively. The primers used for vector construction are listed in Supplementary Table 3. Afterwards, the recombinant vectors and corresponding empty vectors were co-transfected into the yeast strain Y2H Gold, and incubated on the double dropout (DDO) medium (SD/−Leu/-Trp) (#630417, Takara) at 30 °C for 3 days. Then, the monoclonal colonies were spotted on QDO medium. Yeast cells were photographed after 3 days at 30°C to record growth.

In the GST pull-down assay, the LsSP1 was cloned into the pGEX-6P-1 vector (GE Healthcare, Piscataway, USA) for fusion expression with GST, whereas the OsOryzain peptidase domain was cloned into PET-28a (Novagen, Darmstadt, Germany) for fusion expression with His-tag. Empty pGEX-6P-1 was used as the negative control. Later, the proteins were expressed in *E. coli* strain Transetta (#CD801-02, TransGen Biotech, Beijing, China) by induction with 0.1 mM isopropyl β-D-thiogalactoside (IPTG) (A100487, Sango Biotechnology) at 28 °C for 6 h. Afterwards, the GST-LsSP1 and GST proteins were incubated with glutathione-sepharose beads (C600031-0005, Sango Biotechnology) at 4 °C for 2 h. After washing with PBST (consist of PBS and 0.1% Triton-100 (#A110694, Sango Biotechnology) for 4 times, the beads were blocked with 10% fetal bovine serum (FBS) (#F8318, Gibco, New York, USA) for 1 h. Subsequently, His-mOsOryzain was loaded onto the beads and incubated at 4 °C overnight. The beads were further washed with PBST for 4 times,

and the precipitate was added with protein loading buffer. Western-blotting assay was performed as described above. The primary antibody against the His-tag (1:3,000, #MA1-21315, ThermoFisher Scientific) and goat anti-mouse IgG-HRP antibody (1:10,000, #31430, ThermoFisher Scientific) were used.

In the BiFC assay, the LsSP1 was cloned into the pCV-cYFP vector, whereas the mOsOryzain, PLCPs, and LsMLP were cloned into pCV-nYFP vector. Thereafter, the recombinant vectors were transfected into *A. tumefaciens* GV3101 as described above. Then, the *A. tumefaciens* co-transfected with recombinant vectors and corresponding empty vectors were co-infiltrated into *N. benthamiana* leaves. The infiltrated *N. benthamiana* were maintained in a climate chamber for 36-48 h. YFP fluorescence was captured under the Leica SP8 confocal microscope.

In luciferase complementation (LUC) assay, LsSP1 was cloned into pCAMBIA1300-cLUC vector, whereas the mOsOryzain and LsMLP were cloned into pCAMBIA1300-nLUC vector, respectively. The recombinant vectors and corresponding empty vectors were transformed into *A. tumefaciens* GV3101, respectively, which were subsequently co-infiltrated into different areas of the same *N. benthamiana* leaf at a final concentration of OD_{600} =1.0. At 36 h post-infiltration, 0.2 mM LUC substrate was infiltrated into the whole leaves, and images were obtained using a low light cooled CDD imaging apparatus (LUMAZONE SOPHIA2048B, USA).

To investigate the interaction between OsOryzain and salivary sheath, *L. striatellus* samples were allowed to feed on artificial diets for 24 h. Then, the parafilm attached with salivary sheath was collected and washed with PBST thrice. After being fixed in 4% paraformaldehyde for 30 min, the parafilm was incubated with the *N. benthamiana* homogenate containing OsOryzain-GFP or GFP protein at 4 °C for 2 h. Subsequently, the parafilm was washed for 3 times, and GFP fluorescence was observed under Leica SP8 confocal.

***In vitro* protease activity assay**

The EnzChek protease assay kit (#E6638, Molecular Probes, Oregon, USA) was used to evaluate the protease activity of apoplast. Briefly, the rice apoplast was harvested using Buffer B (consist of 0.1 mol/L Tris-HCl, and 0.2 mol/L KCl, pH 7.6). Then, the GST-LsSP1 and GST proteins were purified with glutathione-sepharose beads (C600031-0005, Sango Biotechnology) and washed by PBS using a 3-kDa molecular weight cut-off Amicon Ultra-4 Centrifugal Filter Device. E64

(#E3132, Sigma-Aldrich) and protease inhibitor (PI) cocktail (#A32953, ThermoFisher Scientific) were used as the positive controls. Later, the collected rice apoplast was mixed with different concentrations of E64, PI, GST-LsSP1 and GST proteins, and the mixture was added into the 96-well Immulon plates (ThermoFisher Scientific) containing the BODIPY-FL casein substrate. After reaction for 30 min in dark, fluorescence was measured with a microplate reader (BioTek, Winooski, VT, USA) at the excitation and emission wavelengths of 495 and 530 nm, respectively.

Agrobacterium-mediated plant transformation in *N. benthamiana*

The recombinant expression vectors were transfected into *A. tumefaciens* strain GV3101 by the heat transfer method, and were grown on LB medium containing 50 μg/mL kanamycin and 10 μg/mL rifampicin for approximately 60 h at 28 °C. Later, the colonies containing target vectors were further amplified in LB medium and collected by centrifugation at 2,400 g for 2 min. Subsequently, the agrobacterium was suspended in an induction buffer (10 mM $MgCl_2$, 10 mM MES (pH 5.6), 200 μM Acetosyringone) to OD_{600} =1.0 for 2 h. The wall-associated kinase 2 with an ER retention signal HDEL, C-terminal region of CASP, and vacuolar membrane aquaporinγ-TIP were used as ER, Golgi, and tonoplast markers, respectively[8]. After mixing equal amounts of the selected combinations, the suspension was infiltrated into the approximately 6-week old *N. benthamiana* leaves.

Diaminobenzidine (DAB) staining

The H_2O_2 level in *N. benthamiana* was detected by DAB staining. Briefly, *N. benthamiana* leaf was cut and immersed into 1 mg/mL 3,3'-Diaminobenzidine Tetrahydrochloride (DAB-HCl) (pH 3.8, #D8001, Sigma, St. Louis, MO, USA) for 6 h. Thereafter, the DAB solution was replaced with 100% ethanol and decolored overnight at 65°C. Then, the stained leaf was photographed using a Canon EOS 80D camera (Canon Inc., Tokyo, Japan).

Generation of transgenic *koOry* plants

The *koOry* plants with *OsOryzain* knockout of Nipponbare background were generated using the CRISPR/Cas9 system via the custom service of BioRun Biosciences, Wuhan, China. Briefly, the specific target of *OsOryzain* (5'- CCTACCTCGGCCTCAGGAAC -3') was predicated using CCTop (http://crispr.cos.uni-heidelberg.de/index.html), and ligated into the single guide RNA

(sgRNA) expression cassettes by overlapping PCR. The PCR product was later cloned into the CRISPR/Cas9 vector and introduced into *A. tumefaciens* EHA105. Rice transformation was conducted using the *A. tumefaciens* through callus inoculation and plant regeneration as described above, and hygromycin-resistant transgenic plants were selected. Mutation was determined by PCR with genomic DNA as the template using OsOryzain-specific primers (Supplementary Table 3), followed by Sanger sequencing (YouKang Biotech). Two independent T2 homozygous knockout lines (Supplementary Fig. 17b, c) were used for subsequent experiments. To determine OsOryzain knockout in *koOry* plants, wild type Nipponbare rice plants and *koOry* plants were sprayed with 0.5 μM SA. Samples were collected at 24 h-post treatment.

Supplementary Figures 1-23

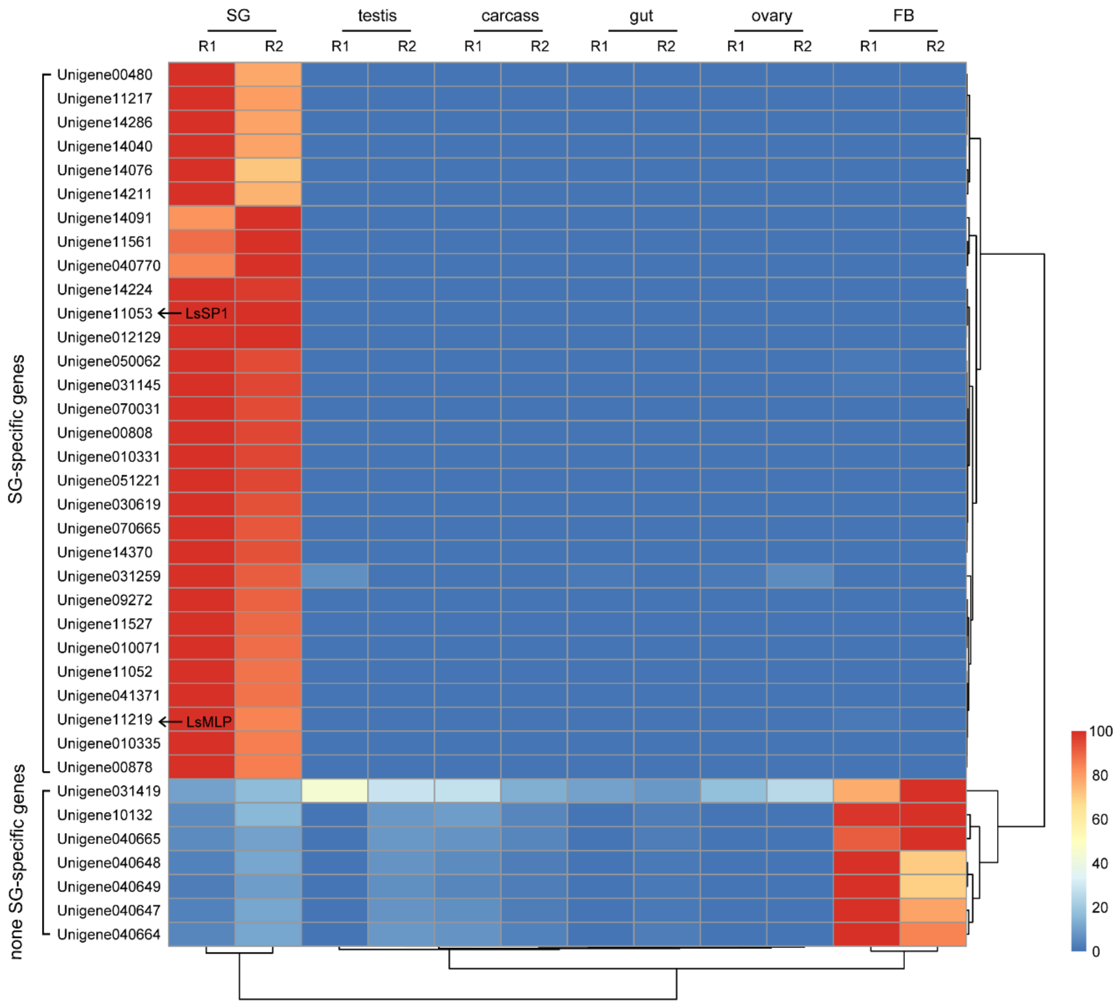

Supplementary Fig. 1. Expression patterns of planthopper-specific genes in different tissues. The expression patterns of planthopper-specific genes in different tissues were analyzed based on the transcripts per million (TPM) expression values, which were generated by analyzing the transcriptomic data of *Laodelphax striatellus* salivary gland (SG), gut, fat body (FB), carcass, testis, and ovary (Supplementary Data 1). The max TPM value of each gene was set as 100. LsSP1 and LsMLP analyzed in this study were indicated by arrows. A total of 30 planthopper-specific genes were found to be specifically expressed in salivary glands. Source data and corresponding sequences are provided in a Source Data file.

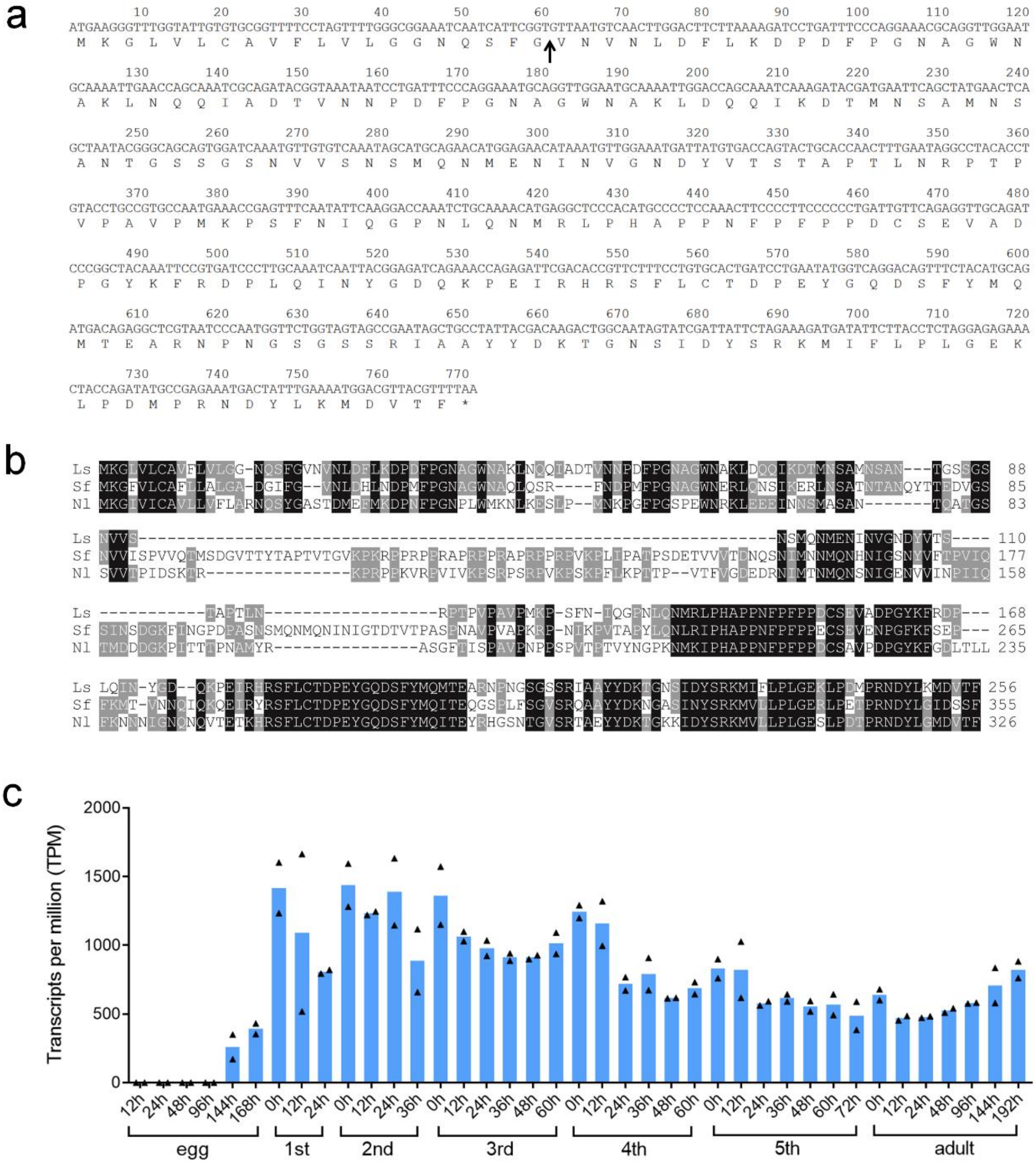

Supplementary Fig. 2. Characteristics of LsSP1. a Nucleic acid and amino acid sequences of LsSP1 (accession number: ON322955). Arrow indicates the signal peptide cleavage site. **b** Amino acid alignment of LsSP1 with its homologous genes in the brown planthopper *Nilaparvata lugens* (Nl, ASL05017) and the white-backed planthopper *Sogatella furcifera* (Sf, ON322954). Black shades indicate the conserved regions in three planthoppers. Ls, *Laodelphax striatellus*. **c** Expression patterns of LsSP1 in *L. striatellus* at different developmental stages. Transcripts per million (TPM) expression values from 39 developmental stages were determined based on the transcriptomic data. Data are presented as mean values (n=2 independent biological replicates). Source data are provided as a Source Data file.

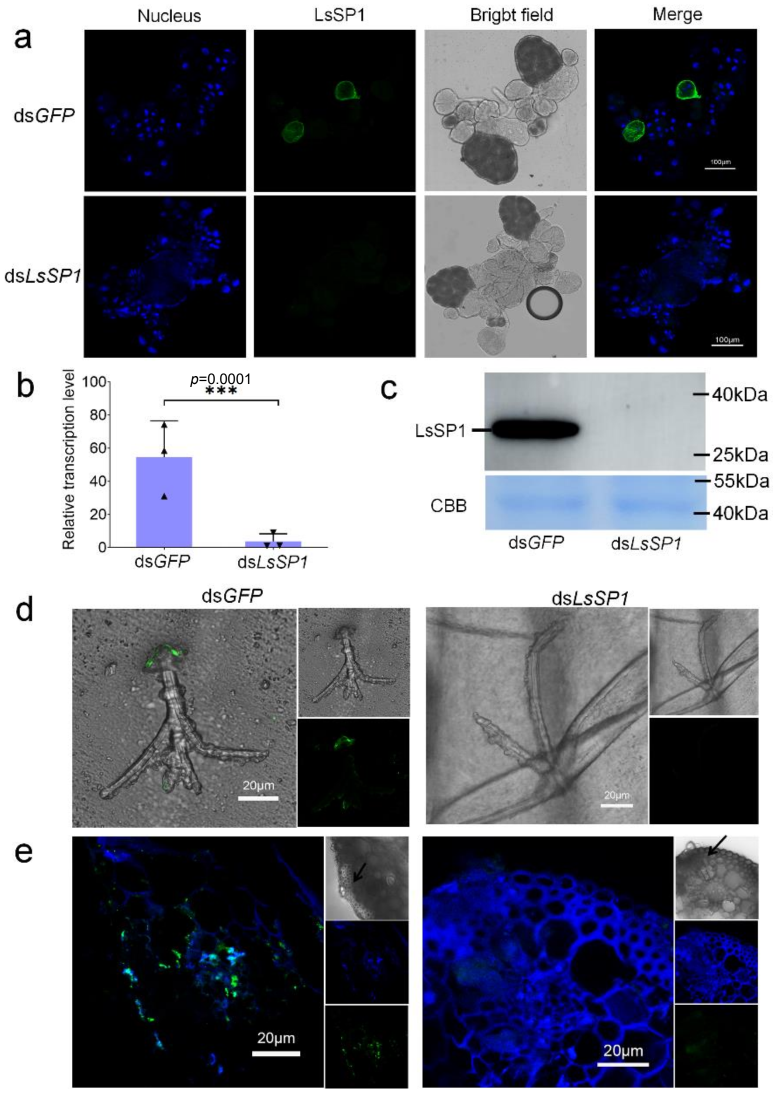

Supplementary Fig. 3. Inhibitory effects of LsSP1. a Immunohistochemical staining of LsSP1. Salivary glands collected from ds*GFP*- and ds*LsSP1*-treated *Laodelphax striatellus* were incubated with anti-LsSP1 serum conjugated with Alexa Fluor™ 488 NHS Ester (green) and were visualized by Leica SP8. The nucleus was stained with DAPI (blue). **b, c** RNAi efficiency was determined by qRT-PCR (**b**) and Western-blotting (**c**) assays. Data are presented as mean values ± SEM (n=3 independent biological replicates). *P*-values were determined by two-tailed unpaired Student's *t*-test. ***$P < 0.001$. Coomassie brilliant blue (CBB) staining was conducted to visualize the amount of sample loading. **d, e** LsSP1 staining of salivary sheath on parafilm (**d**) and in rice tissues (**e**) secreted from ds*GFP*- and ds*LsSP1*-treated *L. striatellus*. Green, LsSP1; blue, nucleus. Experiments in **a, c, d,** and **e** were repeated three times with the similar results. Source data are provided as a Source Data file.

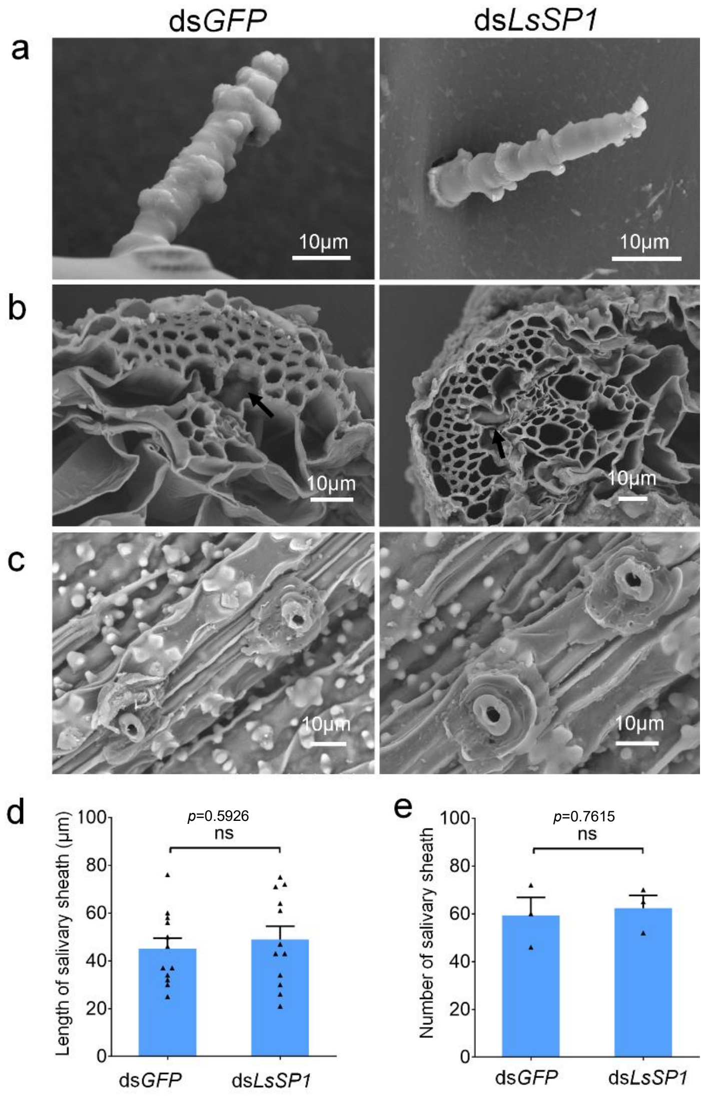

Supplementary Fig. 4. Effects of ds*LsSP1* treatment on salivary sheath formation. *Laodelphax striatellus* were treated with ds*GFP* and ds*LsSP*, respectively. At 4 days later, the insects were fed on rice plants or artificial diets for 24 h. Salivary sheaths on the parafilm (**a**), in rice sheath (**b**), and on rice surface (**c**) were visualized by scanning electron microscopy (SEM). Arrows indicate the salivary sheath in rice sheath. **d** The length of salivary sheath on parafilm secreted from dsRNA-treated *L. striatellus*. **e** The number of salivary sheaths on rice surface secreted from dsRNA-treated *L. striatellus*. Data are presented as mean values ± SEM (n=12 independent biological replicates in **d**; n=3 independent biological replicates in **e**). *P*-values were determined by two-tailed unpaired Student's *t*-test. ns, not significant. Experiments in **a**, **b**, **c** were repeated three times with the similar results. Source data are provided as a Source Data file.

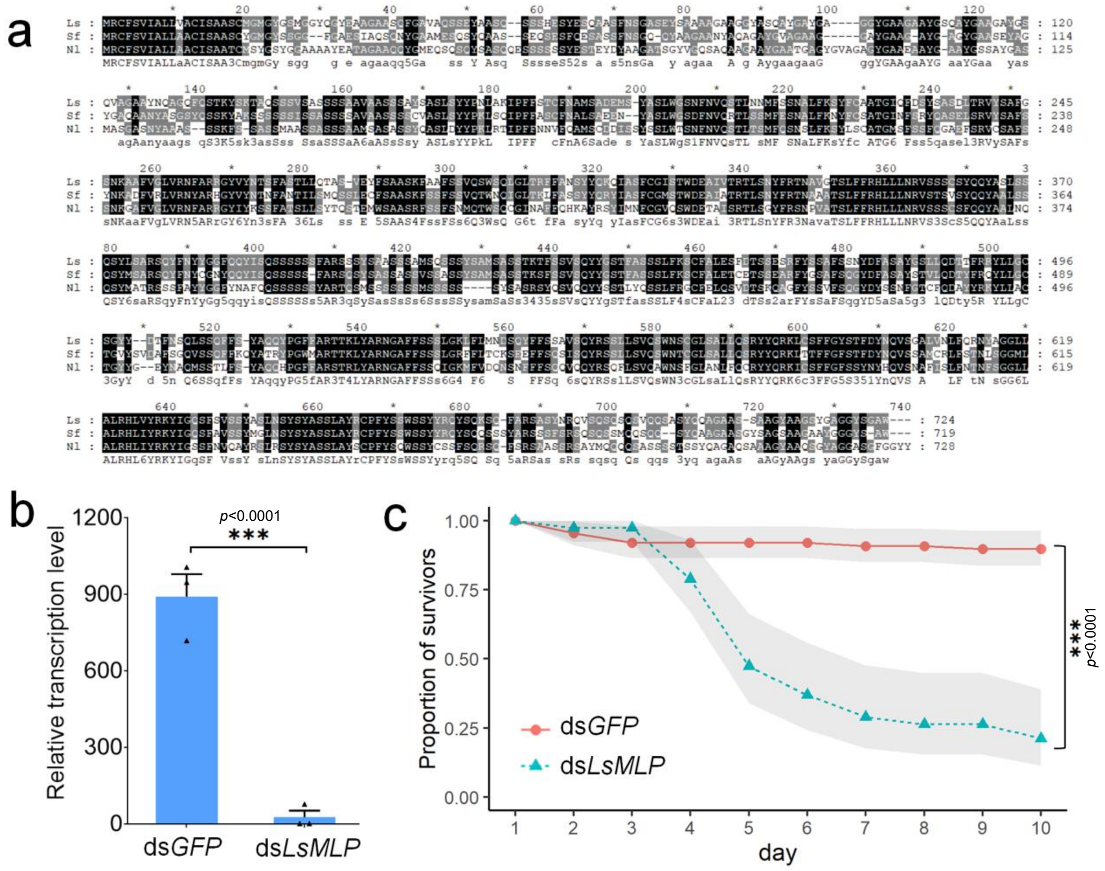

Supplementary Fig. 5. Characteristics and function of LsMLP. a Amino acid alignments of MLPs from *Laodelphax striatellus* (Ls), *Nilaparvata lugens* (Nl), and *Sogatella furcifera* (Sf). Black shades indicate the conserved regions of MLPs in three planthoppers. **b** The RNAi efficiency of *LsMLP* determined by qRT-PCR. Data are presented as mean values ± SEM (n=3 independent biological replicates). *P*-values were determined by two-tailed unpaired Student's *t*-test. ***P < 0.001. **c** Dynamic analysis of the survival rate following ds*GFP* and ds*LsMLP* treatments. Data are presented as mean values ± 95% confidence intervals (displayed in light shades). n = 54 and n = 38 individuals were analyzed in ds*GFP* and ds*LsMLP*, respectively. *P*-values were determined by the log-rank test. ***P < 0.001. Source data are provided as a Source Data file.

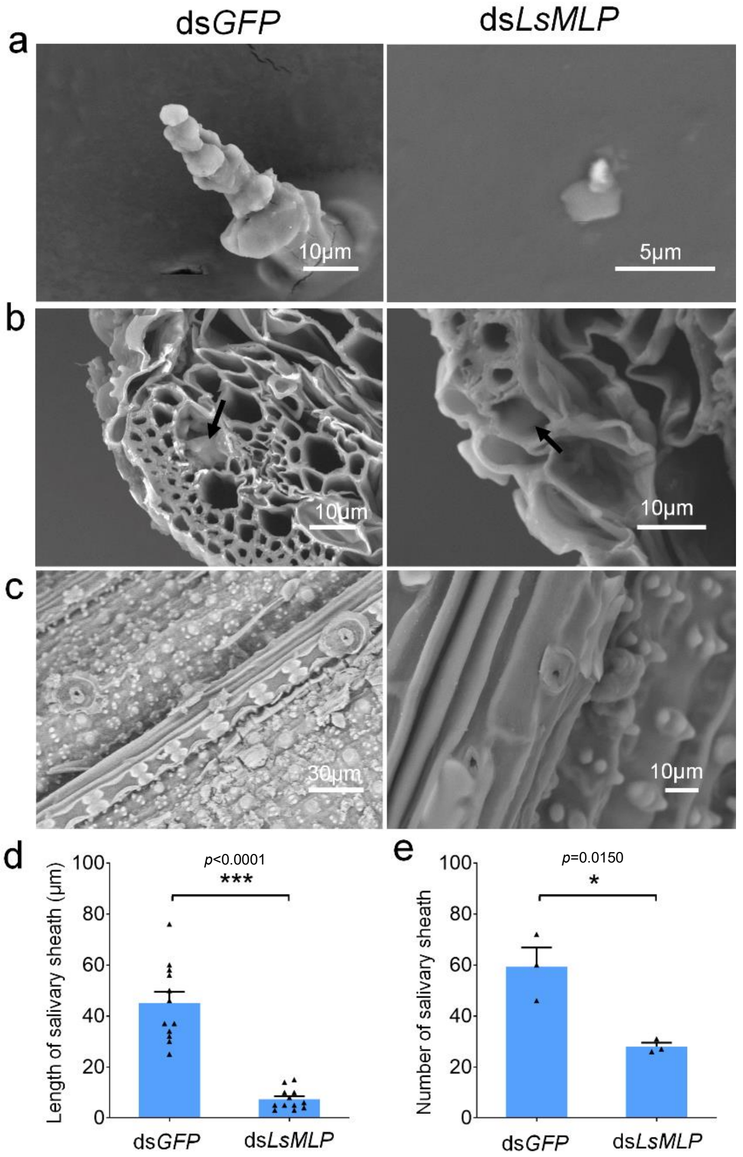

Supplementary Fig. 6. Effects of ds*LsMLP* treatment on salivary sheath formation. *Laodelphax striatellus* were treated with ds*GFP* and ds*LsMLP*, respectively. At 4 days later, the insects were fed on rice plants or artificial diets for 24 h. Salivary sheaths on the parafilm (**a**), in rice sheath (**b**), and on rice surface (**c**) were visualized by scanning electron microscopy (SEM). Arrows indicate the salivary sheath in rice sheath. **d** The length of salivary sheath on parafilm secreted from dsRNA-treated *L. striatellus*. **e** The number of salivary sheaths on rice surface secreted from dsRNA-treated *L. striatellus*. Data are presented as mean values ± SEM (n=12 independent biological replicates in **d**; n=3 independent biological replicates in **e**). *P*-values were determined by two-tailed unpaired Student's *t*-test. *P < 0.05; ***P < 0.001. Experiments in **a**, **b**, **c** were repeated three times with the similar results. Source data are provided as a Source Data file.

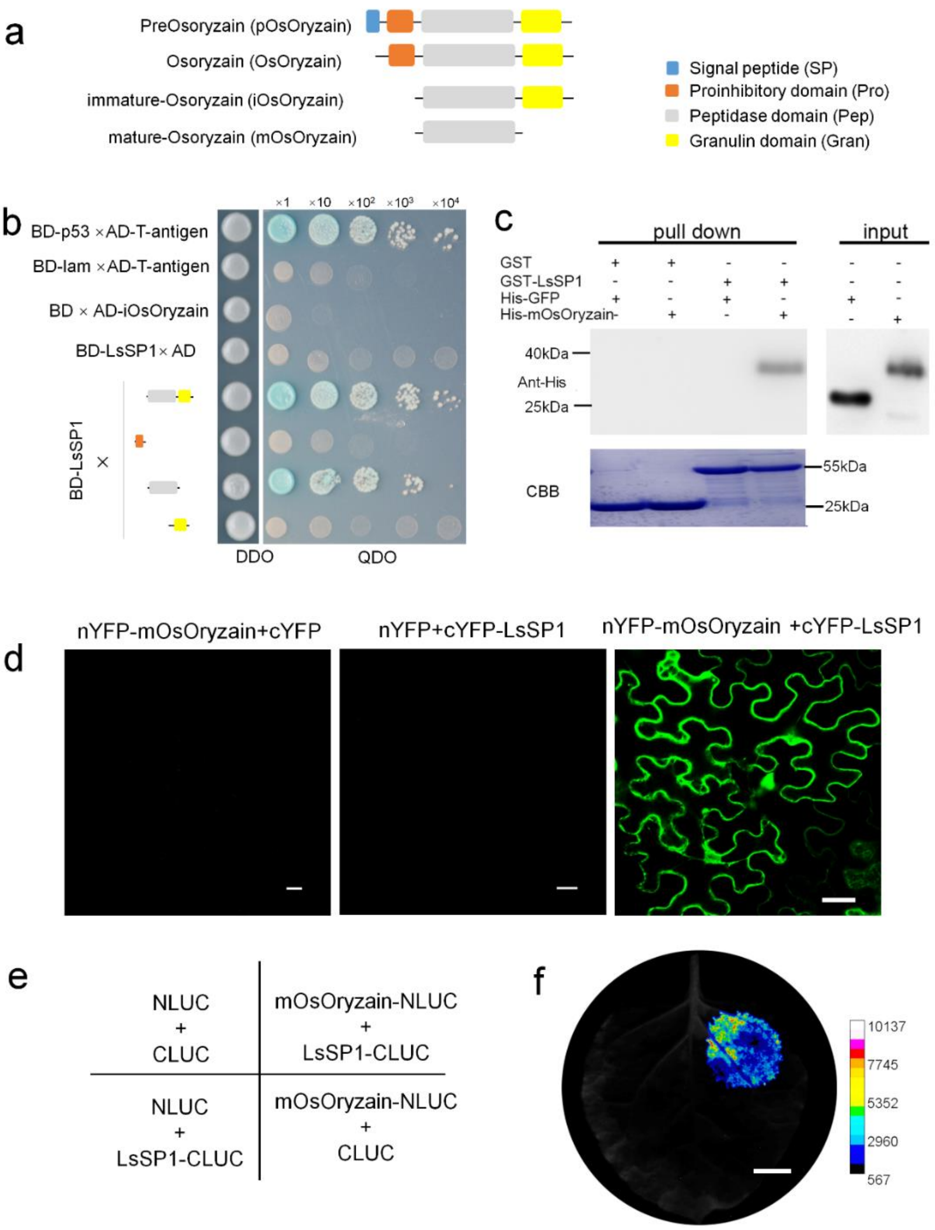

Supplementary Fig. 7. LsSP1 interacts with OsOryzain. a Domain organization of OsOryzain. OsOryzain accumulates in cells as the immature (iOsOryzain) and mature (mOsOryzain) isoforms. **b** Yeast two hybrid assays showing the interaction between LsSP1 and OsOryzain of different isoforms. **c, d** GST-pull down (**c**) and bimolecular fluorescence complementation (BiFC) (**d**) assays confirmed the interaction between LsSP1 and mOsOryzain. Bars=20 µm. **e** The co-expression scheme in *Nicotiana benthamiana* leaves during Luciferase complementation (LUC) assays. **f** Results from LUC assays showing the interaction between LsSP1 and mOsOryzain. Bar=1 cm. Experiments in **c** were repeated two times with the similar results. Experiments in **d** and **f** were repeated three times with the similar results. Source data are provided as a Source Data file.

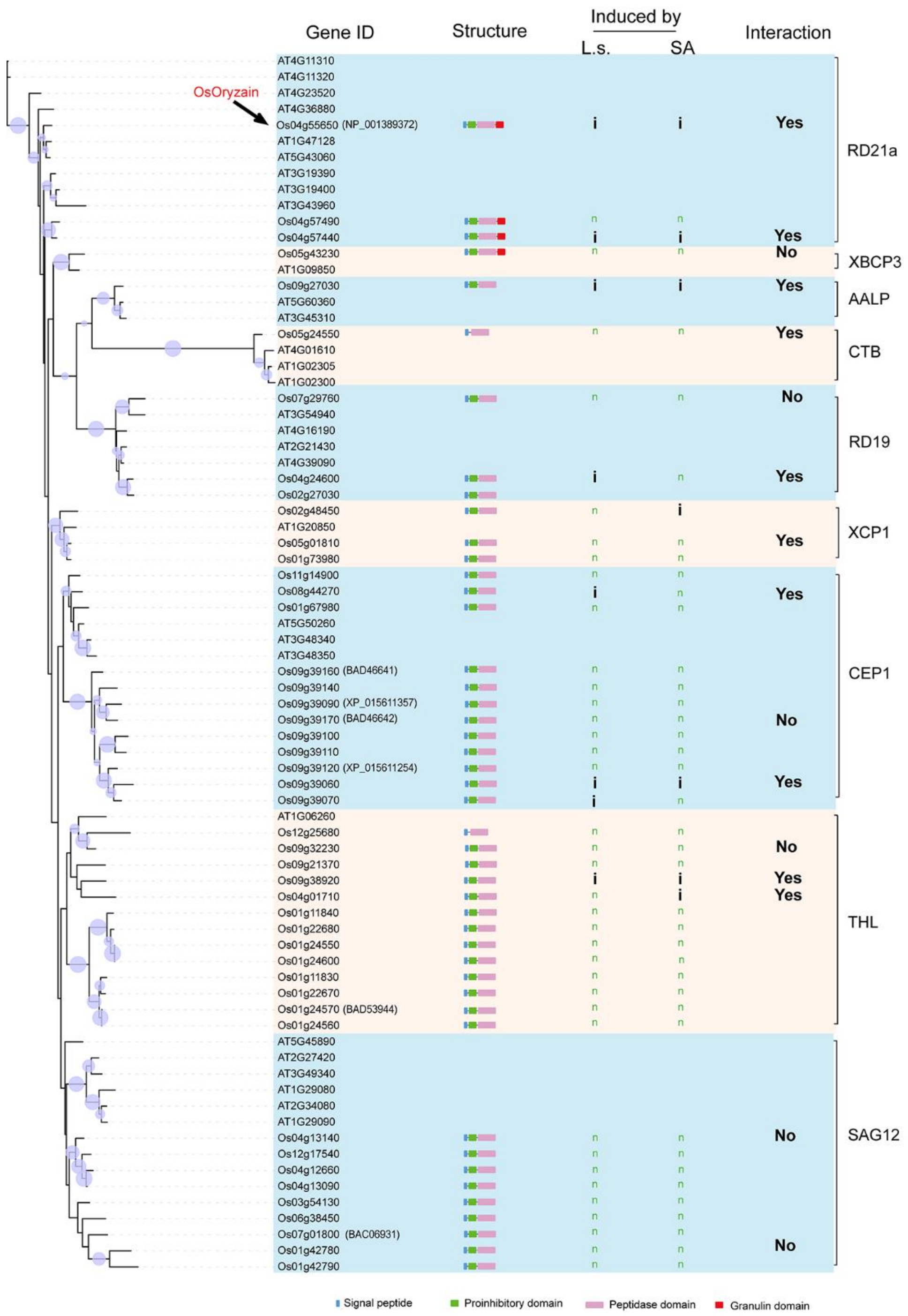

Supplementary Fig. 8. Characteristics of rice PLCPs. Phylogenic and subfamily classifications of canonical PLCPs in *Oryza sativa*. *O. sativa* PLCPs were retrieved from Rice Genome Annotation Project Database. Seven proteins predicted in the Rice Genome Annotation Project Database were incomplete, including Os04g55650 (NP_001389372), Os09g39160 (BAD46641), Os09g39090 (XP_015611357), Os09g39170 (BAD46642), Os09g39120 (XP_015611254), Os01g24570 (BAD53944), and Os07g01800 (BAC06931). The complete coding sequences were retrieved from the NCBI database by BLAST search, and the corresponding GenBank accessions were provided

in the bracket. Phylogenetic analysis of *O. sativa* PLCPs and *A. thaliana* PLCPs was performed by the maximum likelihood method using RAxMLNG with 1000 bootstrap replications. Nodes with bootstrap values >50% are marked with solid blue circles, and the larger circles indicate higher bootstrap values. Arrow indicates the OsOryzain identified by yeast two-hybrid (Y2H) screening. The conserved domains of PLCPs were analyzed by InterPro, and displayed in the "Structure" column. Induction of PLCPs to *Laodelphax striatellus* infestation (L.s.) and salicylic acid treatment (SA) was displayed in Supplementary Fig. 10 and summarized in "L.s." and "SA" columns, respectively. "i" represents a gene induced in at least one time point upon treatments. "n" represents a gene not induced by treatments or gene expression was not detected. Interactions between PLCPs and LsSP1 were displayed in Supplementary Fig. 9 and summarized in "Interaction" column. "Yes" represents a gene that can interact with LsSP1. "No" represents a gene that cannot interact with LsSP1. Source data are provided as a Source Data file.

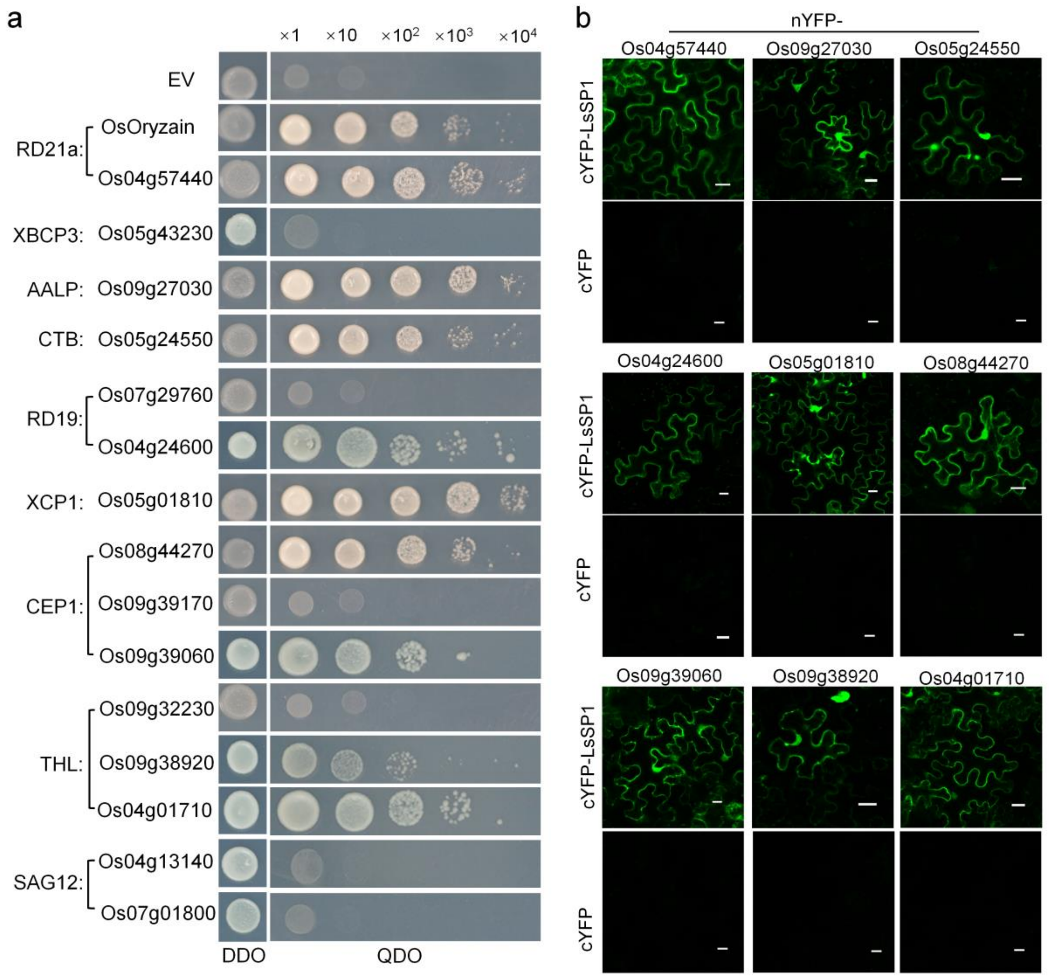

Supplementary Fig. 9. LsSP1 interacts with multiple PLCPs in various subfamilies. a Yeast two hybrid assays showing the interaction between LsSP1 and 15 PLCPs. **b** Bimolecular fluorescence complementation (BiFC) assays confirming the interaction between LsSP1 and 9 PLCPs. Bars=20 μm. Experiments in **b** were repeated three times with the similar results. Source data are provided as a Source Data file.

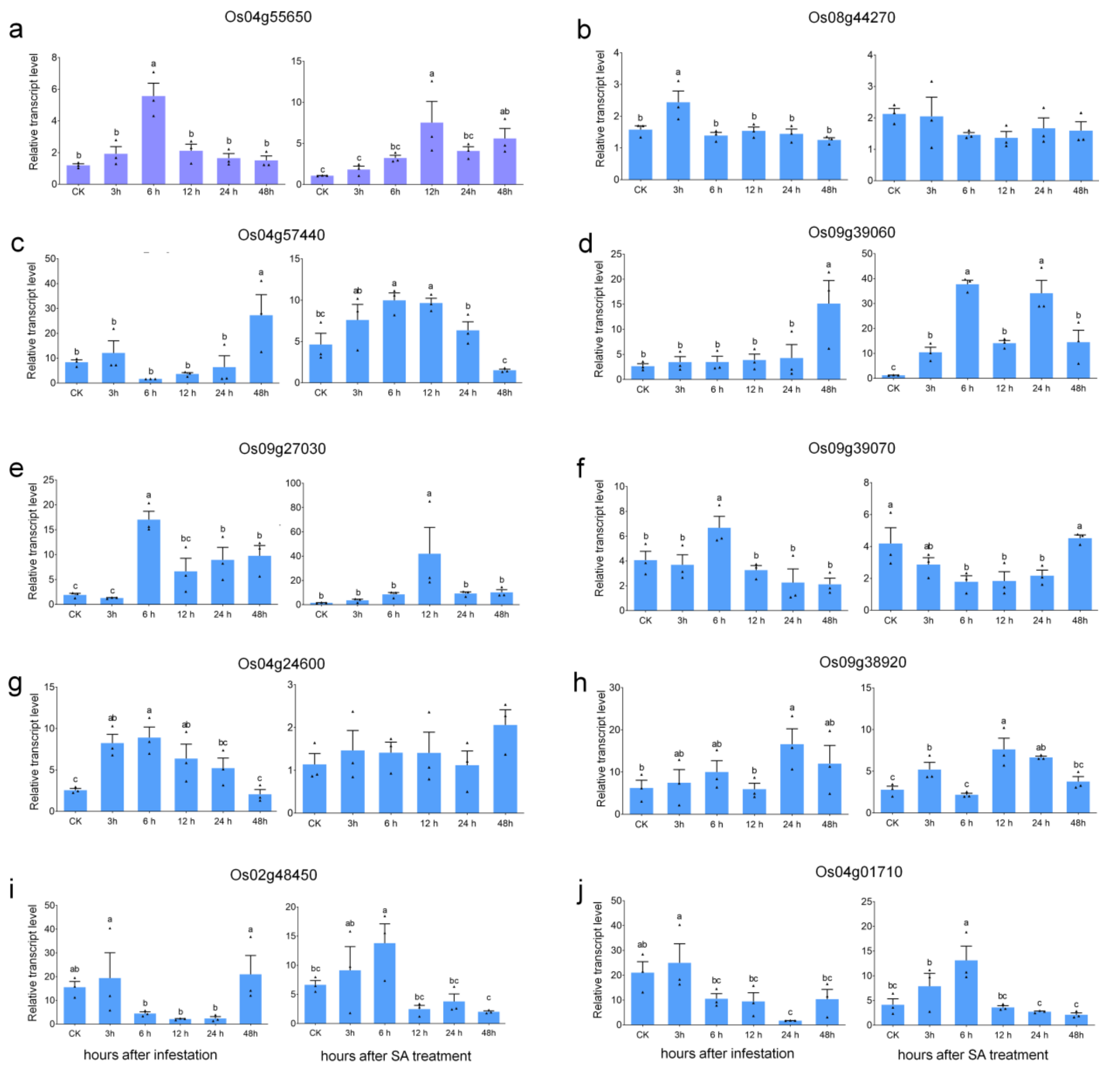

Supplementary Fig. 10. Expression patterns of PLCPs upon *Laodelphax striatellus* infestation and salicylic acid (SA) treatment. Rice plants (cv. ASD7) were treated with *L. striatellus* and SA, and the expression profiles of PLCPs were determined by qRT-PCR. Data are presented as mean values ± SEM (n=3 independent biological replicates). Different lowercase letters indicate statistically significant differences at $P < 0.05$ level according to one-way ANOVA test followed by Tukey's multiple comparisons test. Source data are provided as a Source Data file.

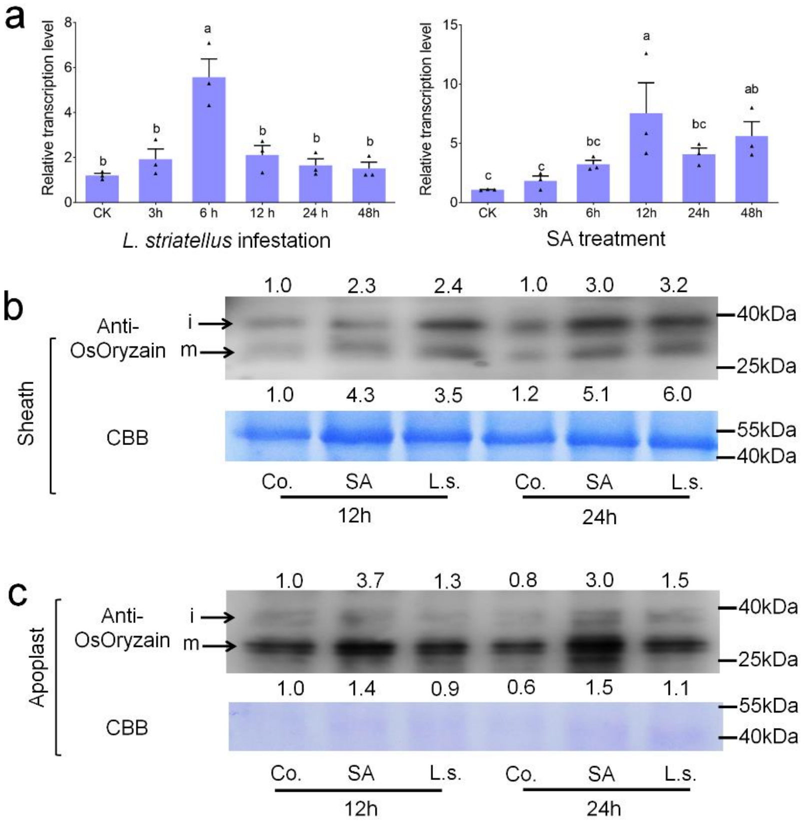

Supplementary Fig. 11. Induction of OsOryzain by different treatments. a Rice plants were treated with *Laodelphax striatellus* or salicylic acid (SA), and the expression profiles of *OsOryzain* were determined by qRT-PCR. Data are presented as mean values ± SEM (n=3 independent biological replicates). Different lowercase letters indicate statistically significant differences at $P <$ 0.05 level according to one-way ANOVA test followed by Tukey's multiple comparisons test. **b, c** The protein levels of OsOryzain in rice sheath (**b**) and rice apoplast (**c**) in response to SA and *L. striatellus* (L.s.) were determined at 12 and 24 h post treatments. Rice plants without treatment were used as controls (Co.). Two bands, corresponding to iOsOryzain (i) and mOsOryzain (m) were detected by Western-blotting assays. Band intensity was measured using ImageJ software, with the band intensity of iOsOryzain and mOsOryzain in untreated sample at 12 h being set at 1.0, respectively. The mean band intensity from 4 biological replicates was displayed near the band. Coomassie brilliant blue (CBB) staining was conducted to visualize the amount of sample loading. The rice variety cv. ASD7 was used. Experiments in **b** and **C** were repeated four times with the similar results. Source data are provided as a Source Data file.

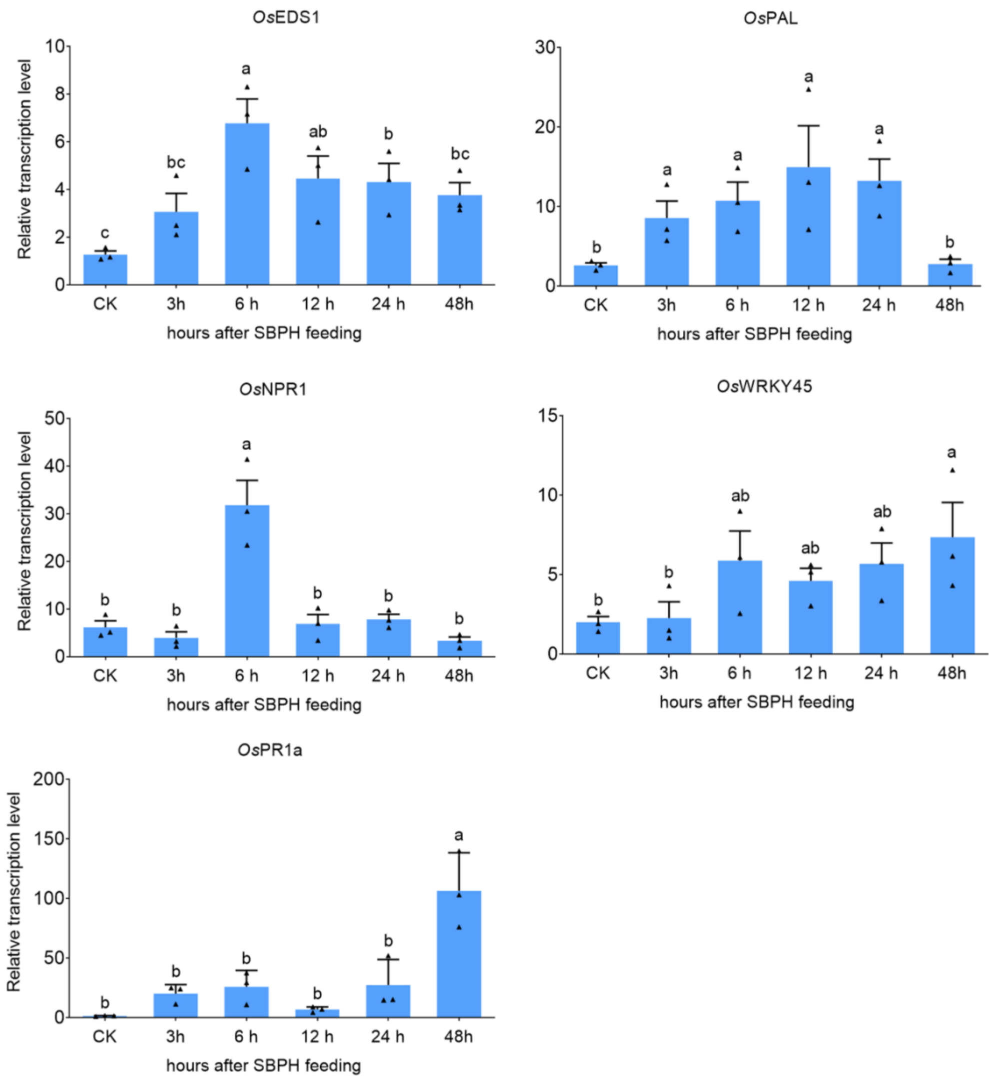

Supplementary Fig. 12. Induction of salicylic acid (SA)-related genes following *Laodelphax striatellus* infestation. Rice plants (cv. ASD7) were infested by *L. striatellus*, and the expression patterns of *enhanced disease susceptibility 1* (*OsEDS1*), *phenylalanine ammonia lyase* (*OsPAL*), *pathogenesis-related 1* (*OsPR1*), *non-expresser of PR genes 1* (*OsNPR1*), and *transcription factor WRKY45* (*OsWRKY45*) were determined by qRT-PCR. Data are presented as mean values ± SEM (n=3 independent biological replicates). Different lowercase letters indicate statistically significant differences at $P < 0.05$ level according to one-way ANOVA test followed by Tukey's multiple comparisons test. Source data are provided as a Source Data file.

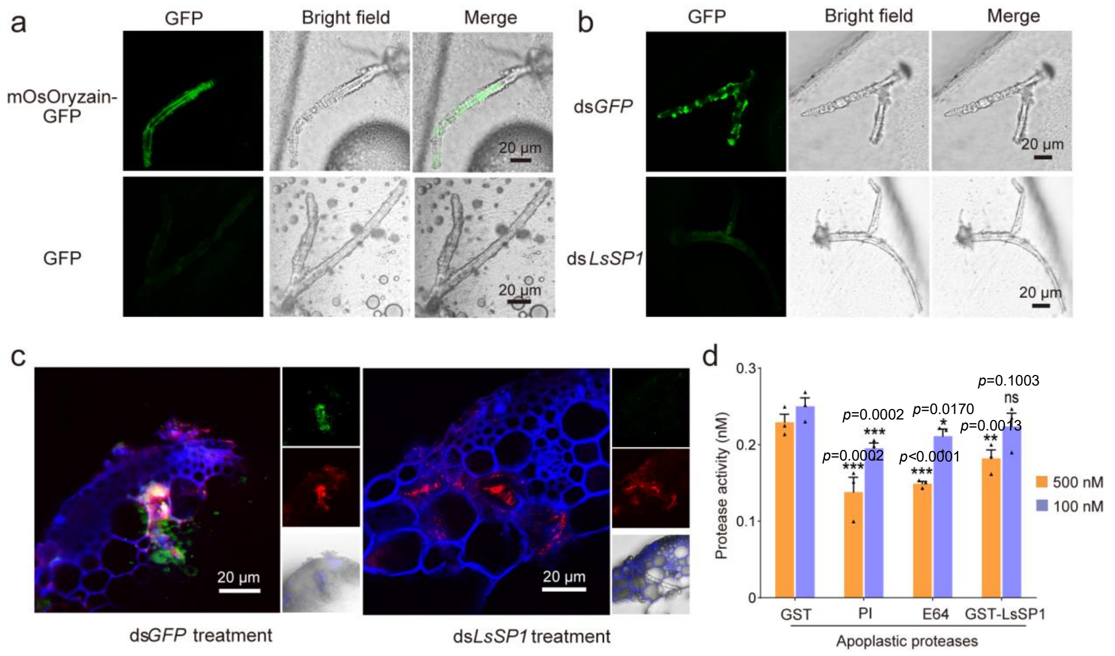

Supplementary Fig. 13. LsSP1 restricts OsOryzain to salivary sheath and inhibits the activity of apoplastic proteases. **a** The binding capacity of salivary sheath to mOsOryzain-GFP or GFP protein. **b** The binding capacity of salivary sheath secreted from dsRNA-treated insects to mOsOryzain-GFP. The salivary sheath was incubated with mOsOryzain-GFP or GFP protein, and the GFP fluorescence was observed. **c** LsSP1 and OsOryzain staining of rice tissues. Rice plants infested by ds*GFP*-(left) and ds*LsSP*- (right) treated insects were cut, and incubated with anti-LsSP1 serum conjugated with Alexa Fluor™ 488 NHS Ester (green) and anti-OsOryzain serum conjugated with Alexa Fluor™ 555 NHS Ester (red). The plant nucleus was stained with DAPI (blue). **d** Proteolytic activity of apoplastic proteases was measured by the digestion of a fluorescent casein substrate in the presence of purified GST protein, proteinase inhibitor cocktail (PI), E64, and purified GST-LsSP1. Fluorescence was measured at the 495/530 nm excitation/emission. Data are presented as mean values ± SEM (n=3 independent biological replicates). *P*-values were determined by two-tailed unpaired Student's *t*-test. *P < 0.05; **P < 0.01; ***P < 0.001; ns, not significant. The rice variety cv. ASD7 was used. Experiments in **a**, **b**, and **c** were repeated three times with the similar results. Source data are provided as a Source Data file.

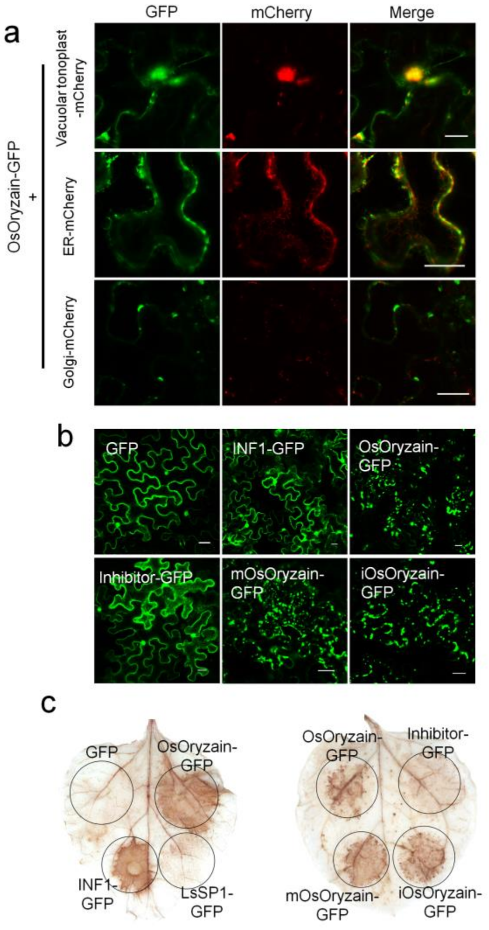

Supplementary Fig. 14. Overexpression of OsOryzain in *Nicotiana benthamiana*. a OsOryzain with a GFP tag, along with the mCherry-tagged organelle marker protein was transiently overexpressed in *N. benthamiana* via *Agrobacterium* infiltration. Vacuolar tonoplast marker, vacuolar membrane aquaporinγ-TIP; endoplasmic reticulum (ER) marker, wall-associated kinase 2 with an ER retention signal HDEL; Golgi marker, C-terminal region of CASP. **b** Subcellular location of OsOryzain and its deletion mutants in *N. benthamiana*. **c** Accumulation of H_2O_2 in *N. benthamiana* revealed by diaminobenzidine (DAB) staining. OsOryzain and its deletion mutants were transiently overexpressed in *N. benthamiana*. *N. benthamiana* overexpressing GFP was used as the negative control. While, *N. benthamiana* overexpressing *Phytophthora infestans* INF1 with a GFP tag was used as the positive control. Bars=20μm. All experiments were repeated three times with the similar results. Source data are provided as a Source Data file.

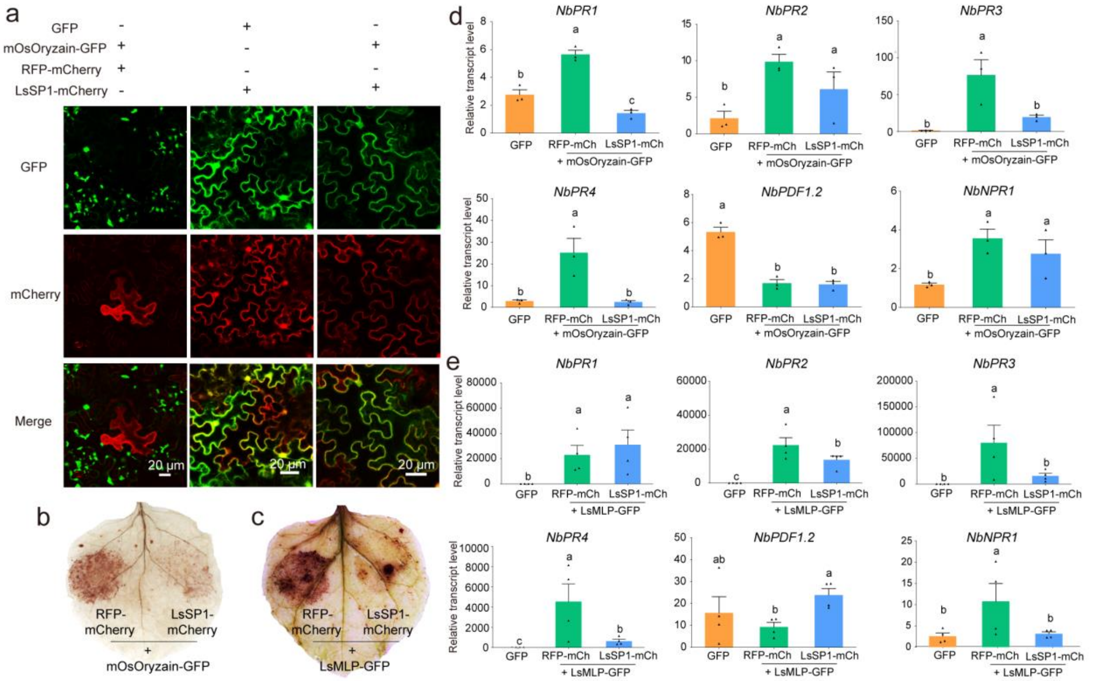

Supplementary Fig. 15. LsSP1 inhibits plant defenses triggered by the overexpression of OsOryzain and LsMLP in *Nicotiana benthamiana*. a LsSP1-mCheery affected the localization patterns of mOsOryzain-GFP in *N. benthamiana*. **b** LsSP1-mCheery overexpression attenuated H_2O_2 accumulation caused by mOsOryzain-GFP overexpression. *N. benthamiana* co-expressing mOsOryzain-GFP and RFP-mCherry was used as a control. **c** LsSP1-mCheery overexpression attenuated H_2O_2 accumulation caused by LsMLP-GFP overexpression. *N. benthamiana* co-expressing LsMLP-GFP and RFP-mCherry was used as a control. **d** Co-expression of LsSP1-mCheery and mOsOryzain-GFP affected the *pathogenesis-related* (*PR*) genes, *plant defensing 1.2* (*NbPDF1.2*), and *non-expresser of PR genes 1* (*NbNPR1*). **e** Co-expression of LsSP1-mCheery and LsMLP-GFP affected the *PR* genes, *NbPDF1.2*, and *NbNPR1*. Data in **d** (n=3 independent biological replicates) and **e** (n=4 independent biological replicates) are presented as mean values ± SEM. mCh, mCherry. Different lowercase letters indicate statistically significant differences at $P < 0.05$ level according to one-way ANOVA test followed by Tukey's multiple comparisons test. Experiments in **a**, **b** and **c** were repeated three times with the similar results. Source data are provided as a Source Data file.

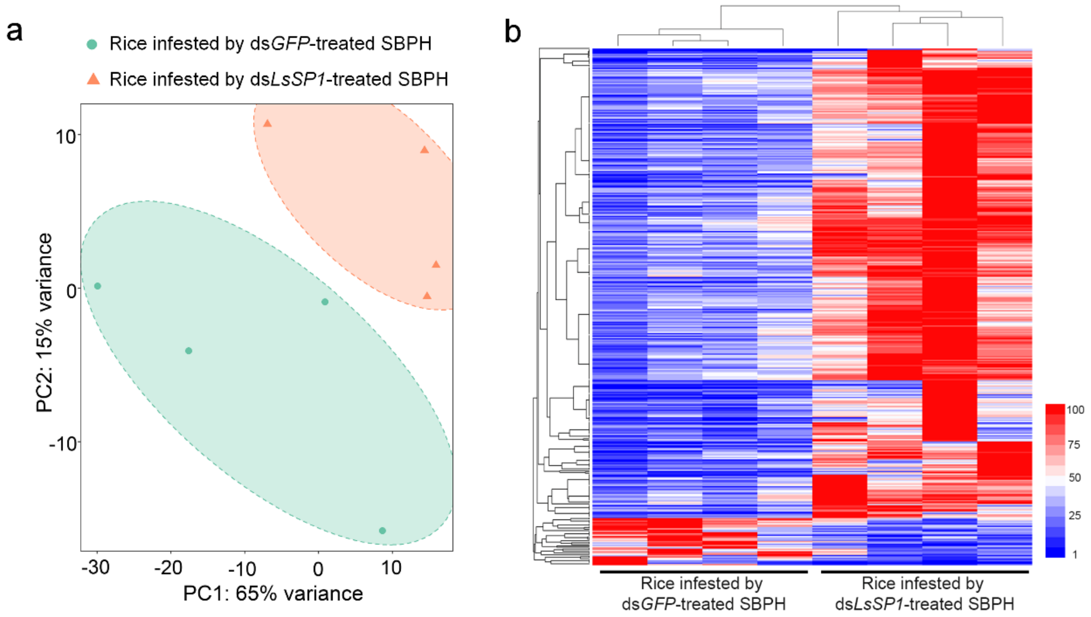

Supplementary Fig. 16. Overview of the influence of dsRNA-treated *Laodelphax striatellus* on rice plants. a Principal component analysis (PCA) of gene expression patterns in rice plants infested by ds*GFP*- and ds*LsSP1*-treated *L. striatellus*. The first two principal components (PC1 and PC2) based on transcriptomic results are shown. **b** Overview of differentially expressed genes (DEGs) identified between rice plants infested by ds*GFP* and ds*LsSP1*-treated *L. striatellus*. The expression level of DEGs were analyzed based on the transcripts per million (TPM) expression values. The max TPM value of each gene was set as 100. There were 90.9% (368/405) of DEGs upregulated in plants infested by ds*LsSP1*-treated *L. striatellus*. Source data are provided as a Source Data file.

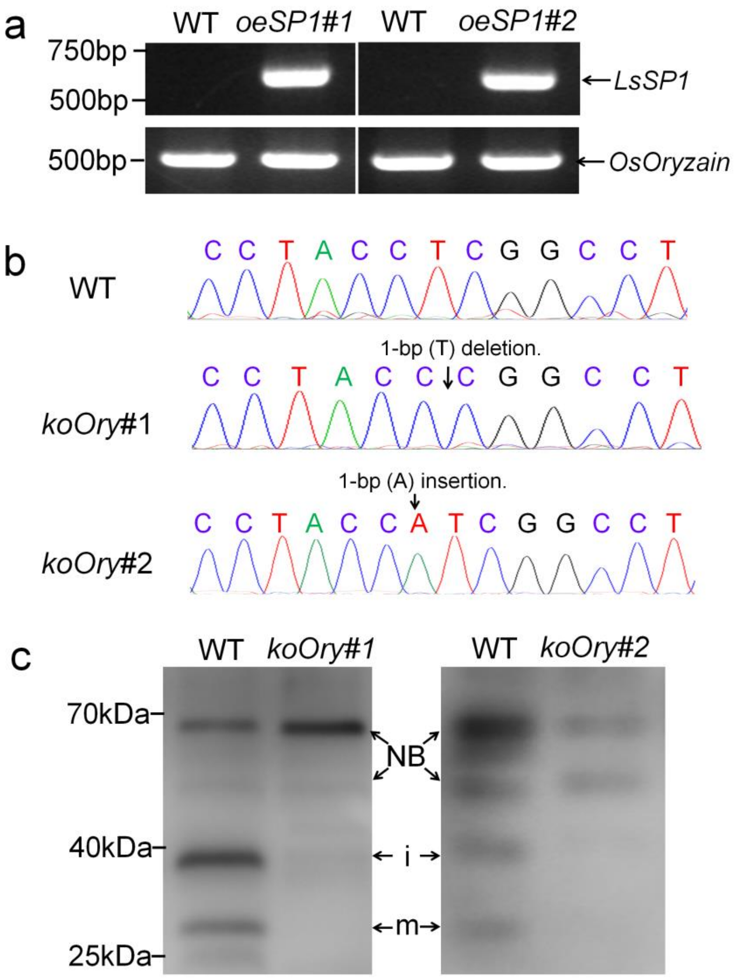

Supplementary Fig. 17. Characteristics of transgenic rice plants. a Expression of *LsSP1* and *OsOryzain* in two independent *oeSP1* plants. **b** Mutation of OsOryzain in two independent *koOry* plants. *koOry*#1 plants have a frame shift mutation caused by a 1-bp deletion. *koOry*#2 plants have a frame shift mutation caused by a 1-bp insertion. **c** Protein levels of OsOryzain in WT and two *koOry* plants. iOsOryzain (i) and mOsOryzain (m) cannot be detected in *koOry*#1 and *koOry*#2 plants. NB, none-specific binding site. Source data are provided as a Source Data file. Experiments in **a** and **c** were repeated three times with the similar results.

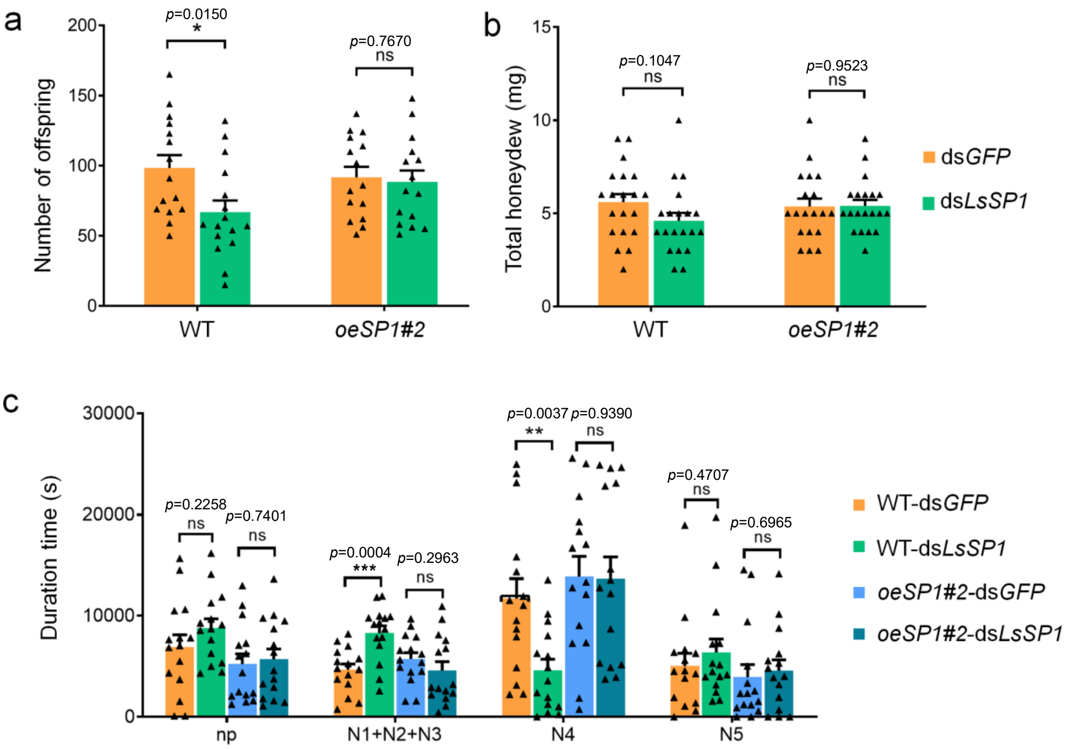

Supplementary Fig. 18. Influence of *Laodelphax striatellus* infestation on wild type (WT) and *oeSP1*#2 plants. a-c Comparison of fecundity (**a**), honeydew excretion (**b**), and electrical penetration graph (EPG) parameters (**c**) between ds*GFP*-treated and ds*LsSP1*-treated *L. striatellus* on WT and *oeSP1*#2 plants. *P*-values were determined by two-tailed unpaired Student's *t*-test. * P < 0.05; **P < 0.01; ***P < 0.001; ns, not significant. Data are presented as mean values ± SEM. For fecundity and honeydew analysis, n=20 independent biological replicates; for EPG analysis, n=15 independent biological replicates. All EPG recordings were performed for 8 h. N1+N2+N3, pathway duration; N4, phloem sap ingestion; N5, xylem sap ingestion; np, nonpenetration. Nipponbare rice plants and transgenic plants of Nipponbare background were used. Source data are provided as a Source Data file.

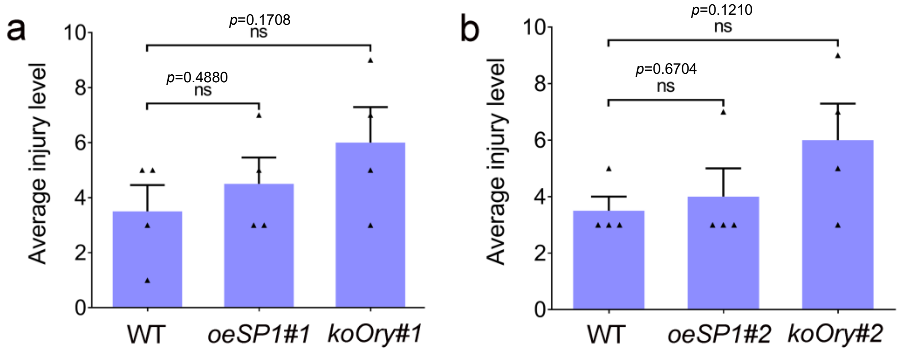

Supplementary Fig. 19. Comparison of *Laodelphax striatellus* resistance in WT, *oeSP1*, and *koOry* plants. Five rice seedlings of comparison group 1 (WT, *oeSP1#1* and *koOry#1*) (**a**) and comparison group 2 (WT, *oeSP1#2* and *koOry#2*) (**b**) at 4-5 leaf stage were grown in a plastic cup. The plants were infested with 4th instar *L. striatellus* nymphs at ten insects per seedling. After 20 days, the injury level of rice plants was checked. The identification standard of Wu et al (1986) was used to calculate the average injury level (Supplementary Table 4). Data are presented as mean values ± SEM (n=4 independent biological replicates). *P*-values were determined by two-tailed unpaired Student's *t*-test. ns, not significant. Source data are provided as a Source Data file.

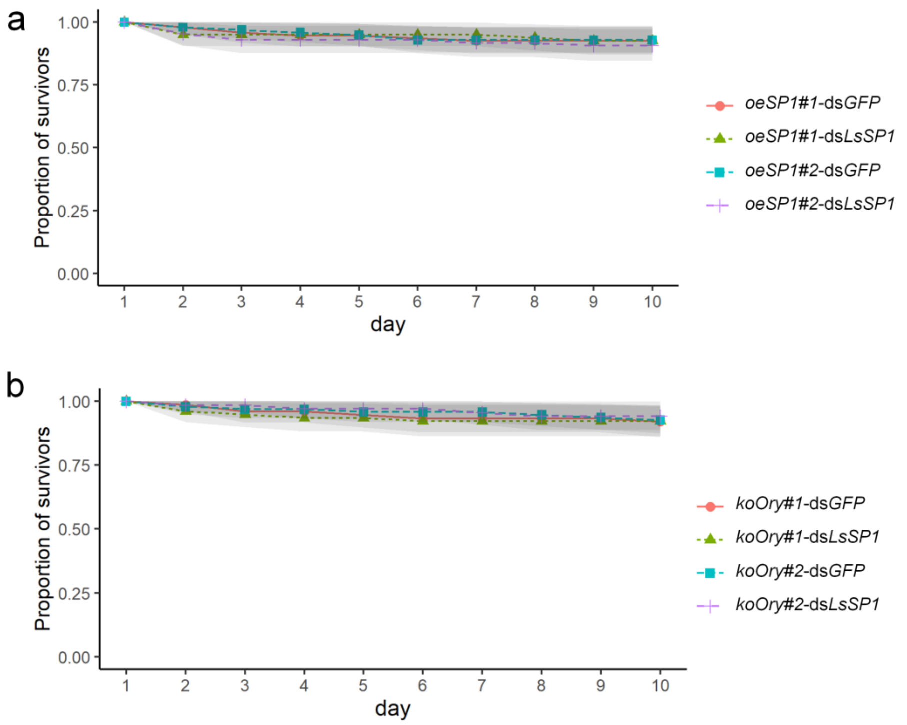

Supplementary Fig. 20. Comparison of survival rate between ds*GFP*-treated and ds*LsSP1*-treated *Laodelphax striatellus* on *oeSP1* and *koOry* plants. a *L. striatellus* survival rates on *oeSP1*#1 and *oeSP1*#2 plants. n= 85, 80, 96, and 82 individuals in *oeSP1*#1-ds*GFP*, *oeSP1*#1-ds*LsSP1, oeSP1*#2-ds*GFP*, and *oeSP1*#2-ds*LsSP1*, respectively. **b** *L. striatellus* survival rates on *koOry*#1 and *koOry*#2 plants. n= 75, 76, 94, and 67 individuals in *koOry*#1-ds*GFP*, *koOry*#1-ds*LsSP1, koOry*#2-ds*GFP*, and *koOry*#2-ds*LsSP1*, respectively. Data are presented as mean values ± 95% confidence intervals (displayed in light shades). Source data are provided as a Source Data file.

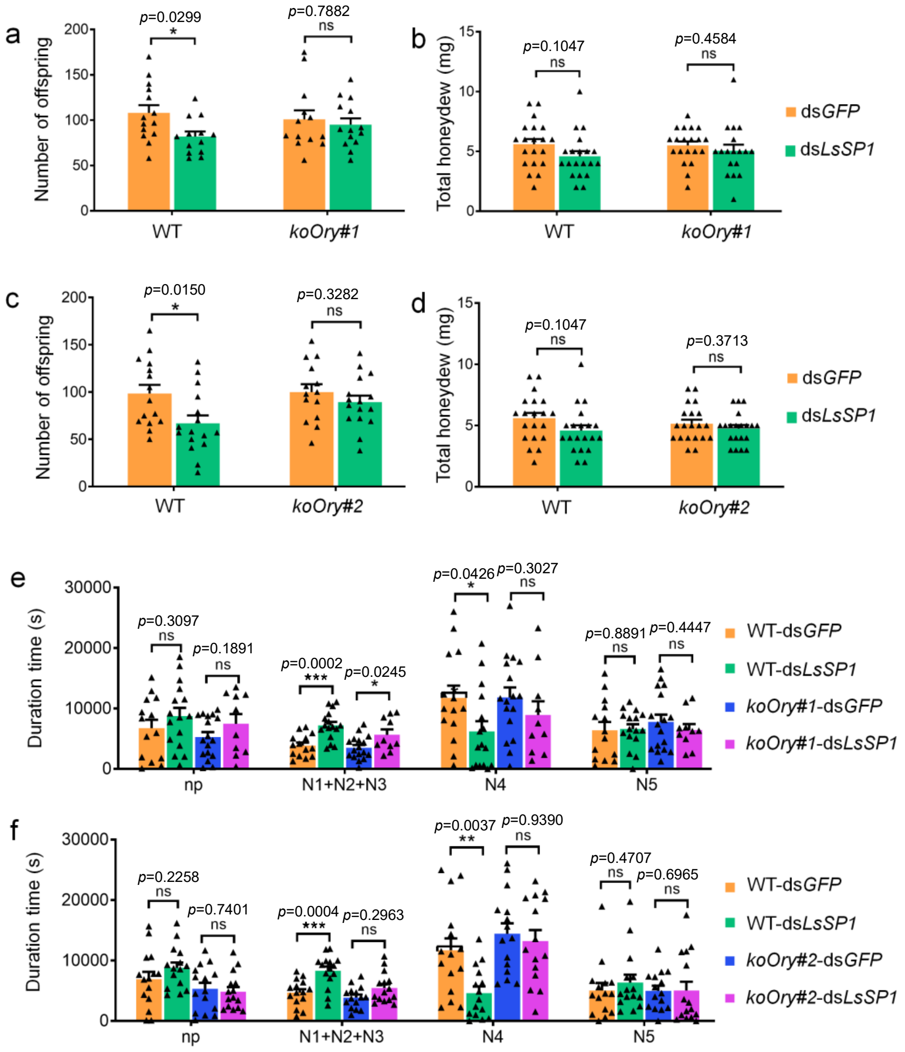

Supplementary Fig. 21. Influence of *Laodelphax striatellus* infestation on wild type (WT) and *koOry* plants. Comparison of fecundity (**a, c**), honeydew excretion (**b, d**), and electrical penetration graph (EPG) parameters (**e, f**) between ds*GFP*-treated and ds*LsSP1*-treated *L. striatellus* on WT and *koOry* plants. The results of comparison group 1 (WT and *koOry#1*) were presented in **a**, **b**, and **e**, while the results of comparison group 2 (WT and *koOry#2*) were presented in **c**, **d**, and **f**. *P*-values were determined by two-tailed unpaired Student's *t*-test. * $P < 0.05$; ** $P < 0.01$; *** $P < 0.001$; ns, not significant. Data are presented as mean values ± SEM. For fecundity and honeydew analysis, n=20 independent biological replicates all treatment (**a**, **b**, **c**, and **d**). In comparison group 1, n=14 (WT-ds*GFP*), n=16 (WT-ds*LsSP1*), n=17 (*koOry*#1-ds*GFP*), and n=10 (*koOry*#1-ds*LsSP1*) independent biological replicates for EPG analyses (**e**). In comparison group 2, n=15 independent biological replicates in each treatment for EPG analyses (**f**). Source data are provided as a Source Data file.

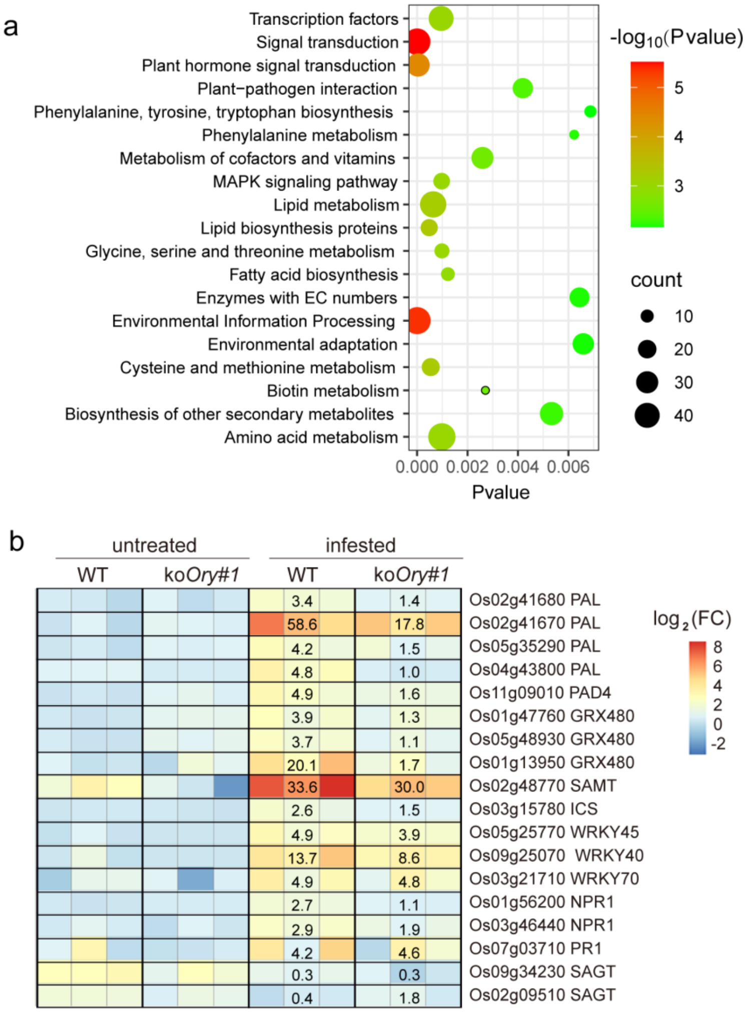

Supplementary Fig. 22. Analysis of *koOry#1* plants that untreated or infested by ds*LsSP1*-treated *Laodelphax striatellus*. a Kyoto Encyclopedia of Genes and Genomes (KEGG) pathway enrichment analysis of DEGs specifically identified in WT plants, but not in *koOry#1* plants. Enriched *P*-values were calculated according to one-sided hypergeometric test using TBtools software. **b** The impacts of *L. striatellus* infestation on salicylic acid (SA) marker genes in WT and *koOry#1* plants. PAL, phenylalanine ammonia lyase; PAD4, phytoalexin deficient 4; GRX480, glutaredoxin GRX480; SAMT, SA methyl transferase; ICS, isochorismate synthase; WRKY, transcription factor WRKY; NPR1, non-repressor of pathogenesis-related protein 1; PR1, pathogenesis-related 1; SAGT, SA glucosyl transferase. Nipponbare rice plants and transgenic plants of Nipponbare background were used. Source data are provided as a Source Data file.

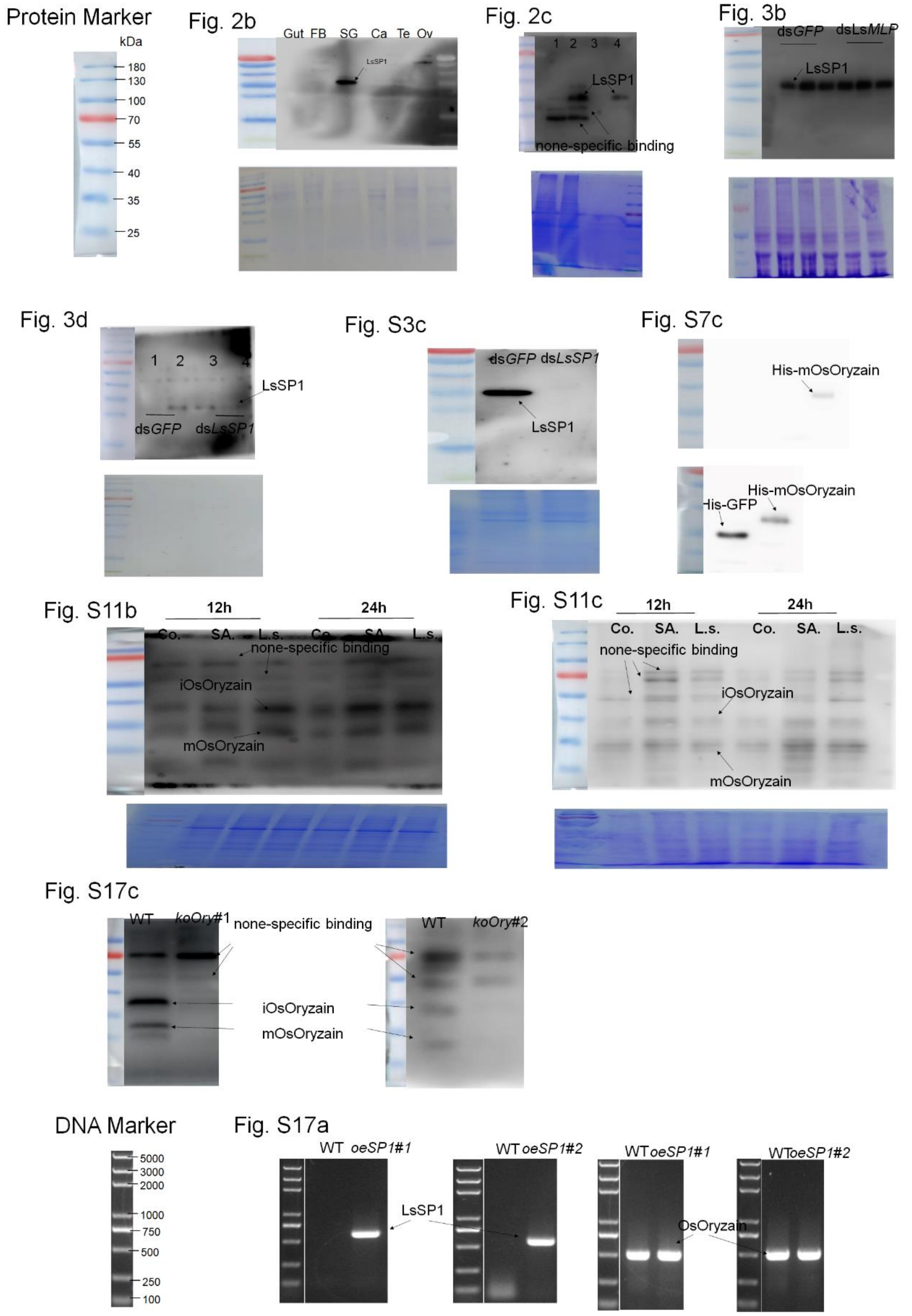

Supplementary Fig. 23. Original images for blots and gels in Figures and Supplementary Figures.

Supplementary Tables 1-4

Supplementary Table 1. Proteins from a rice cDNA library screened by yeast two hybrid (Y2H) using LsSP1 as a bait

GenBank Accession	Annotation	Number of colonies
NP_001389372.1	oryzain alpha precursor	3
KAF2924946.1	hypothetical protein DAI22_06g016200	3
XM_015785635	putative receptor-like protein kinase	2
XP_015639150.1	SNF1-related protein kinase regulatory	2
XP_015632933.1	polypyrimidine tract-binding protein	2
NP_001390973.1	alpha-galactosidase	1
NP_001390734.1	alpha-amylase	1

Supplementary Table 2. Salicylic acid (SA) biosynthesis and SA response gene analyzed in this study

Gene (Abbreviation)	ID
Phytoalexin deficient 4 (PAD4)	Os11g09010
Isochorismate synthase (ICS)	Os09g19734, Os03g15780
Phenylalanine ammonia lyase (PAL)	Os02g41680, Os02g41670, Os05g35290, Os04g43800, Os02g41650
glutaredoxin GRX480 (GRX480)	Os01g47760, Os05g48930, Os01g13950, Os01g27140
SA glucosyl transferase (SAGT)	Os09g34250, Os09g34214, Os04g12980
Non-repressor of pathogenesis-related protein 1 (NPR1)	Os01g09800, Os01g56200, Os03g46440
transcription factor WRKY (WRKY)	Os05g25770, Os03g21710, Os09g16510
SA methyl transferase (SAMT)	Os01g50610, Os02g48770, Os06g13350
Pathogenesis-related 1 (PR1)	Os07g03710, Os07g03730, Os07g03740, Os07g03750

Supplementary Table 3. Primers used in this study

Genes	Forward primer (5'-3')	Reverse primer (5'-3')
**Primers used in qPCR**		
L. striatellus GAPDH	GTGTTGACTACATGGTCTACT	GCTCACTGAATACCTGGATT
L. striatellus actin	AATCGTAAGAGACATCAAGGAG	AGGCAATTCGTAGGACTTCT
LsSP1	GAGATCAGAAACCAGAGATTC	GATACTATTGCCAGTCTTGTC
LsMLP	CTATTGTTACCAGAACCTTGTC	GATGATGATGATGATGACTGAC
O. sativa actin	CAGCACATTCCAGCAGAT	CAGCACATTCCAGCAGAT
O. sativa UBQ10	TGGTCAGTAATCAGCCAGTTTGG	GCACCACAAATACTTGACGAACAG
OsOryzain	CATCAACAATGGTGGAATCG	TGGTGTTACATCTTCGTAGC
OsAALP	CTTGTTCGTCCAGTTAGTGTT	GAGTTCTTGATGAGCCAGTAG
N. benthamiana PR1	TGAGATGTGGGTCGATGAGA	CGAGTTACGCCAAACCACTT
N. benthamiana PR2	TAGAGAATACCTACCCGCCC	GAGTGGAAGGTTATGTCGTGC
N. benthamiana PR3	TGGGGTTATTGCTGGCTTAG	GGGTCATCCAAAACCAGAGA
N. benthamiana PR4	GGCCAAGATTCCTGTGGTAGAT	CACTGTTGTTTGAGTTCCTGTTCCT
N. benthamiana PDF1.2	TGGCAAAATCTATGCGCTTT	ATCCTTCGGTCAAACAGACG
N. benthamiana NPR1	TGCTCTCCATTATGCTGTAG	GCACTGTGTATCCTCTTGAA
O. sativa EDS1	CATTCCAAGAACGAGGACACTG	CAAGACTCAAGGCTAGAACCGA
O. sativa PAL	GCACATCTTGGAGGGAAGCT	GCGCGGATAACCTCAATTTG
O. sativa NPR1	TGAGAGTCTACGAGGAAGGTTGC	CGTTGTCTTTCAGGAGGTGGAT
O. sativa WRKY45	GGACGCAGCAATCGTCCGGG	CGGAAGTAGGCCTTTGGGTGC
O. sativa PR1	GGTGTCGGAGAAGCAGTGGTA	GCGAGTAGTTGCAGGTGATGAAG

O. sativa WRKY55	CTACAGATGTGGTTACAGTGA	TCTTCTTCCTCTTCCTTCAAG
O. sativa Pik-2	TAGGAGGTCTGTTAGCCAATA	TAGTGATGTTACGAAGGTCAG
O. sativa CDPK13	TCATCCTCTACATCCTCCTCT	GATGCTCAAGAACTTGTTTGG
O. sativa response regulator 9	GTAGTTACCACTGTTGATTCC	CGATTCCTTGATCTTCTTGAG
MAPKKK16	GGAAACGATCAACGAATCTTC	TTAATGAGACCCAAGCAGAAC
Os09g32230	TTCATGTTCCAGCTCTACG	CCACGAGTTCTTCACGAT
Os01g24550	GCAGGAGTTCCAGTTCTACAA	GAGTTCTTGGCGATCCAGTAC
Os04g01710	GCTCCTACTCGCAATAATATG	GTCCATCATCATCATCATCAG
Os04g13140	GGCACTTCCAGTTCTACAAGG	CGAGTTCTTCATCAGCCAGTA
Os04g12660	GACATGACATTCCAGTTCTAC	GAGTTCTTCAGCAACCAATAC
Os04g13090	TACAAGGATGCTACTGAGAAG	CTAACCGTGCTAGGAATGAAT
Os04g57440	GAACTTGTAGAATGCTCAACC	CTTCAGTATCAATGCCTCCAT
Os07g01800	TCAAGAACCAACGATCCTGC	GTACTGGAACGCATTGTCGA
Os01g22670	TAATATTGCAGTCGTCGTCCT	CATCCACTCCTCGAACATCTG
Os01g42780	TCAACCAGTTCTCCGACCTCA	CTGCGTTGGTTCTTGACCT
Os01g24570	GGCGTTCCAGTTCTACAAGTC	CCTTCTCCAGCAGGATATAGC
Os01g73980	CCAGTGAATTGTCCATAGTTG	GGTGATCTTCTTGTTCTCCTC
Os01g42790	TGTTCGAGCAGGAGGAGAAG	TTGCCGGTCTCGATCTTGATC
Os01g11840	TAAGCCTAGTTACTACCTAAGC	GAACAAGAACCTCATCTCCTT
Os01g22680	GCTTGTCTTGTCCATCACTTG	GAACATGAACCTCATCTCCCT
Os01g24560	GGCGTTCCAGTTCTACAAGTC	GTCCTTCTCCAGCAGGATGTA

Os01g67980	GACCAGTCCTTCCAGTTCTAC	CGAGTTCTTGACGATCCAGTA
Os01g11830	GGCTATTGCTGTCATTCTCTT	CATCCACTCCTCGAACATCTG
Os01g24600	CTTCTTCCAAGCCTCTCCTTC	GAACATGAACCTCAGCTCCTT
Os03g54130	AAGCTGATCGACTCGTTCAAC	GCTGAAGTTCTCGTACAGGAA
Os02g27030	ATGGTGGATTGATGACTAACG	AGAGACAACACTGAAGTTCTG
Os02g48450	GCAAGTTGGTATCACTATCAG	GTAGTCTTCCTCTGTGTAGAT
Os09g39140	GAAGTCATTAGTAGTGGTGTTG	CGAACCTACTCTTCTTATCCT
Os09g38920	GACAACTTCCAGCACTACAGG	ACGAGTTCTTGATGATCCAGTA
Os09g39100	ATCACAATCAGCGATGAGTTC	ATGCCACAGATACCATTCTTG
Os09g39160	GCTACGAATTCATGATCTACC	CGAGTTCTTGACTATCCAGTA
Os09g39060	AAGAAGGCATTAGTAGTAGGG	CTTCTTGTTGAACTCGCTGAC
Os09g39090	GTGGGTTATGGTGTGAATACT	TTAGTAGGAGGCGATGATAGT
Os09g39070	CATGTGGAATCTGTACGATAG	GTCATACCTTCCTTCTTGTTG
Os09g39120	TGTGACACTGAACAACACTAAG	CAGCATCAGCAATAACAGAGG
Os09g39110	GTGTCCGTTATCATCCAAATC	GAGTTCTTGACGATCCAGTAT
Os09g21370	GTTCTACAAGGGTGGCGTTTA	ATGAGTTCTTGATGGTCCAGTA
Os06g38450	TGCTATCCTCCTCATCATCAT	TTGGTCCTGAATACCTCGAAT
Os12g17540	AAGTATTGGCTGGTGAAGAAC	CTACTCAGTAGGGTAGGAAGG
Os12g25680	GTTGAGCTTGTTGAGAAGTTC	CTCTTCGGCAGAATATCATCA
Os05g43230	CCGAACAGGAACTAATAGACT	CAACAGCCTGAAGTAACATATC
Os05g24550	CACCTTCCTGTTGTTGTTAGT	CATCAGCGATCTTGGATAAGT
Os11g14900	GATGTAACGATTGATGGCTAC	GAGTTATACCGTAACCGACAG

Os04g24600	TACAGAGCGAGAAAGATTACC	GGAGATCACACTGAAGTTCTT
Os05g01810	CCAAGATTACATCATCGTGAAG	TCTTGTTGATGCCACATAGAC
Os08g44270	GTACTGGATCGTCAGGAACTC	CTCCTCGTCATCATCATCTTC
Os09g27030	CTTGTTCGTCCAGTTAGTGTT	GAGTTCTTGATGAGCCAGTAG
Os09g39170	CAGCAGTACATCGTCAAGAAC	TTGTTCACCCACTTTACATCG
Os04g57490	ACATCATGTCGATCATCAGGTA	GAACTTGAGGTTGTCCCAGAA
Os07g29760	CCTACGCCTACCTGATGA	CGAGTTCTTGATGATCCAGTA
**Primers used in double stranded RNA synthesis**		
GFP	TAATACGACTCACTATAGGGAGAATGAGTAAAGG AGAAGAACTTTTC	TAATACGACTCACTATAGGGAGATTTGTATAGTTC ATCCATGCCATGT
LsSP1	TAATACGACTCACTATAGGGGTTAATGTCAACTTG GACTTC	TAATACGACTCACTATAGGGCGTAACGTCCATTTT CAAATAG
LsMLP	TAATACGACTCACTATAGGGATGGGCATGGGATAC GGTAGT	TAATACGACTCACTATAGGGCTTGTTAGATCCAAA AGCGCTG
**Primers used in binary vector construction**		
GFP-LsSP1	GACGAGCTGTACAAGGGTACCATGGTTAATGTCA ACTTGGACTTC	GCGGACTCTAGTTCATCTAGACGTAACGTCCATTT TCAAATAG
GFP- LsMLP	SACGAGCTGTACAAGGGTACCATGGGCATGGGAT ACGGTAGT	GCGGACTCTAGTTCATCTAGATTACCAAGCTCCGG AGTAGCC
OsOryzain	ACGAGCTGTACAAGGGTACCATGGACATGTCGAT CGTGTCGTAC	GCGGACTCTAGTTCATCTAGAAGCGCTGCTCTTCT TGCCGTTG
mOsOryzain	ACGAGCTGTACAAGGGTACCATGTACCGGGACAC	GCGGACTCTAGTTCATCTAGAGTTCTCGCCCTTCT

	CTACCTCGG	TCAGAGG
OsOryzain-Inhibitor	ACGAGCTGTACAAGGGTACCATGGACATGTCGATCGTGTCGTAC	GCGGACTCTAGTTCATCTAGAGTACTCCTCGTTGGTGAGGTC
iOsOryzain	ACGAGCTGTACAAGGGTACCATGTACCGGGACACCTACCTCGG	GCGGACTCTAGTTCATCTAGATCAAGCGCTGCTCTTCTTGCC
Flag/mCheery-LsSP1	CGACGACAAGACCGTCACCATGGTTAATGTCAACTTGGACTTC	GAGGAGAAGAGCCGTCGCGTAACGTCCATTTTCAAATAG
mCheery-RFP	CTTCGACGACAAGACCGGGCCCATGGCCTCCTCCGAGAACGTCA	AGTGAGGAGAAGAGCCGGGCCCACAGGAACAGGTGGTGGCGG
**Primers used in Y2H vector construction**		
AD-LsSP1	GTACCAGATTACGCTCATATGGTTAATGTCAACTTGGACTTC	CAGCTCGAGCTCGATGGATCCCGTAACGTCCATTTTCAAATAG
AD-LsMLP	GTACCAGATTACGCTCATATGATGGGCATGGGATACGGTAGT	CAGCTCGAGCTCGATGGATCCTTACCAAGCTCCGGAGTAGCC
AD-OsOryzain	GTACCAGATTACGCTCATATGGACATGTCGATCGTGTCGTAC	CAGCTCGAGCTCGATGGATCCTCAAGCGCTGCTCTTCTTGCC
AD-OsOryzain-Inhibitor	GTACCAGATTACGCTCATATGGACATGTCGATCGTGTCGTAC	CAGCTCGAGCTCGATGGATCCGTACTCCTCGTTGGTGAGGTC
AD-mOsOryzain	GTACCAGATTACGCTCATATGTACCGGGACACCTACCTCGG	CAGCTCGAGCTCGATGGATCCGTTCTCGCCCTTCTTCAGAGG
AD-OsOryzain-Granulin	GTACCAGATTACGCTCATATGCCTCTGAAGAAGGGCGAGAAC	CAGCTCGAGCTCGATGGATCCTCAAGCGCTGCTCTTCTTGCC

AD- Os04g57440	GTACCAGATTACGCTGATCTCACCAACGAGGAGTTC	CAGCTCGAGCTCGATGGATCCTGCGGTGTTCAGCTTAGCGAG
AD- Os05g01810	GTACCAGATTACGCTCATATGGAGTTCAAGGCGACCTACCTC	CAGCTCGAGCTCGATGGATCCGTTGTCCTTGGTGGGGTAAG
AD- Os05g24550	GTACCAGATTACGCTCATATGACGGCTGCACGGAATCCATAC	CAGCTCGAGCTCGATGGATCCTCAAACTATGGCCGTTCCGAC
AD- Os09g39170	GTACCAGATTACGCTCATATGCTCAACAAGTTCGCCGACATG	CAGCTCGAGCTCGATGGATCCCTAGTAGGATGCGATAGCAG
AD- Os09g32230	GTACCAGATTACGCTCATATGCAGATTTCAAATACCTGCTCC	CAGCTCGAGCTCGATGGATCCTCACATGACGGGGTAGCTC
AD- Os07g29760	GTACCAGATTACGCTCATATGGTTGGCGACGACGTCCGGAG	CAGCTCGAGCTCGATGGATCCAGAGCCGGTAGTAGCCCTGC
AD- Os08g44270	GTACCAGATTACGCTCATATGTTCGGCGACATGACCGCCGAC	CAGCTCGAGCTCGATGGATCCGTGCTCCTCGTCATCATCATC
AD- Os09g27030	GTACCAGATTACGCTCATATGTTCCGGATCTTCTCCGAGAGC	CAGCTCGAGCTCGATGGATCCCAAGTAGCAATACCGCACATG
AD-Os04g01710	GTACCAGATTACGCTCATATGGAAACGCCGTTCACCGACCTC	CAGCTCGAGCTCGATGGATCCTCACCGAATGGTGGGAAAGGTC
AD-Os04g13140	GTACCAGATTACGCTCATATGACGCGGACGGGGCTCAGG	CAGCTCGAGCTCGATGGATCCTCACGCCGTCGGGTAGGAGG
AD-Os01g42780	GTACCAGATTACGCTCATATGTTCGCGCAGACGCACCTCG	CAGCTCGAGCTCGATGGATCCGTCCATGGTCGGGTAGAAGG

AD-Os09g38920	GTACCAGATTACGCTCATATGTTCATCGCCATGTACACCGC	CAGCTCGAGCTCGATGGATCCTCACATGAGCGGGAATGACG
AD-Os09g39060	GTACCAGATTACGCTCATATGGAGGAGTTCGTCGCCAAGTAC	CAGCTCGAGCTCGATGGATCCTCGATTAATTACCTCCTTGTAG
AD-Os04g24600	GTACCAGATTACGCTCATATGTTCCGCGACCGCTTCCTCGG	CAGCTCGAGCTCGATGGATCCGGAGGTATGGATGGCAGTCAC
AD-Os05g43230	GTACCAGATTACGCTCATATGCTCAACGCCTTCGCGGACCTCA	CAGCTCGAGCTCGATGGATCCGCAATAGGTAAGAAGGCTGCA
BK-LsSP1	TCAGAGGAGGACCTGCATATGGTTAATGTCAACTTGGACTTC	CCGCTGCAGGTCGACGGATCCCGTAACGTCCATTTTCAAATAG
BK-LsMLP	TCAGAGGAGGACCTGCATATGATGGGCATGGGATACGGTAGT	CCGCTGCAGGTCGACGGATCCTTACCAAGCTCCGGAGTAGCC
**Primers used in prokaryotic expression vector construction**		
LsSP1	TTCCAGGGGCCCCTGGGATCCGTTAATGTCAACTTGGACTTC	GTCACGATGCGGCCGCTCGAGCGTAACGTCCATTTTCAAATAG
mOsOryzain	CAGCAAATGGGTCGCGGATCCTACCGGGACACCTACCTCGG	GTGGTGGTGGTGGTGCTCGAGGTTCTCGCCCTTCTTCAGAGG
**Primers used in BIFC vector construction**		
CY-LsSP1	CTGTACAAGTCCGGAGTCGACATGGTTAATGTCAACTTGGACTTC	GATCGGGGAAATTCGAGCTCCGTAACGTCCATTTTCAAATAG
NY-Os04g13140	ATCGAGGACTCCGGAGTCGACATGACGCGGACG	GATCGGGGAAATTCGAGCTCTCACGCCGTCGGGT

	GGGCTCAGG	AGGAGG
NY-Os04g01710	ATCGAGGACTCCGGAGTCGACATGGAAACGCCGTTCACCGAC	GATCGGGGAAATTCGAGCTCTCACCGAATGGTGGGAAAGGTC
NY-Os09g38920	ATCGAGGACTCCGGAGTCGACATGTTCATCGCCATGTACACC	GATCGGGGAAATTCGAGCTCTCACATGAGCGGGAATGACG
NY-Os09g39060	ATCGAGGACTCCGGAGTCGACATGGAGGAGTTCGTCGCCAAG	GATCGGGGAAATTCGAGCTCTCGATTAATTACCTCCTTGTAG
NY-Os04g24600	ATCGAGGACTCCGGAGTCGACATGTTCCGCGACCGCTTCCTC	GATCGGGGAAATTCGAGCTCGGAGGTATGGATGGCAGTCAC
NY-Os05g43230	ATCGAGGACTCCGGAGTCGACATGCTCAACGCCTTCGCGGACC	GATCGGGGAAATTCGAGCTCGCAATAGGTAAGAAGGCTGCA
NY-Os05g01810	ATCGAGGACTCCGGAGTCGACATGGAGTTCAAGGCGACCTACCTC	GATCGGGGAAATTCGAGCTCGTTGTCCTTGGTGGGGTAAG
NY-Os05g24550	ATCGAGGACTCCGGAGTCGACATGACGGCTGCACGGAATCCATAC	GATCGGGGAAATTCGAGCTCTCAAACTATGGCCGTTCCGAC
NY-Os08g44270	ATCGAGGACTCCGGAGTCGACATGTTCGGCGACATGACCGCCGAC	GATCGGGGAAATTCGAGCTCGTGCTCCTCGTCATCATCATC
NY-Os09g27030	ATCGAGGACTCCGGAGTCGACATGTTCCGGATCTTCTCCGAGAGC	GATCGGGGAAATTCGAGCTCCAAGTAGCAATACCGCACATG
NY-Os04g24600	ATCGAGGACTCCGGAGTCGACATGTTCCGCGACCGCTTCCTC	GATCGGGGAAATTCGAGCTCGGAGGTATGGATGGCAGTCAC

NY-Os09g39060	ATCGAGGACTCCGGAGTCGACATGGAGGAGTTC GTCGCCAAG	GATCGGGGAAATTCGAGCTCTCGATTAATTACCTC CTTGTAG
NY-Os09g38920	ATCGAGGACTCCGGAGTCGACATGTTCATCGCCA TGTACACC	GATCGGGGAAATTCGAGCTCTCACATGAGCGGGA ATGACG
NY-Os04g01710	ATCGAGGACTCCGGAGTCGACATGGAAACGCCG TTCACCGAC	GATCGGGGAAATTCGAGCTCTCACCGAATGGTGG GAAAGGTC
NY-LsSP1	ATCGAGGACTCCGGAGTCGACATGGGCATGGGAT ACGGTAGT	GATCGGGGAAATTCGAGCTCTTACCAAGCTCCGG AGTAGCC
NY-mOsOryzain	ATCGAGGACTCCGGAGTCGACATGTACCGGGACA CCTACCTCGG	GATCGGGGAAATTCGAGCTCAGTTCTCGCCCTTC TTCAGAGG
**Primers used in LUC vector construction**		
NLUC-mOsOryzain	ACGAGCTCGGTACCCGGGATCCATGTACCGGGAC ACCTACCTCGG	GACGCGTACGAGATCTGGTCGACGTTCTCGCCCT TCTTCAGAGG
CLUC-LsSP1	GTACGCGTCCCGGGGCGGTACCATGGTTAATGTC AACTTGGACTTC	GAACGAAAGCTCTGCAGGTCGACTTACGTAACGT CCATTTTCAAATAG
CLUC- LsMLP	ACGAGCTCGGTACCCGGGATCCATGGGCATGGGA TACGGTAGT	GACGCGTACGAGATCTGGTCGACCCAAGCTCCGG AGTAGCCACC
**Primers used in analyzing transgenic plants**		
LsSP1-specific	GTTAATGTCAACTTGGACTTC	CGTAACGTCCATTTTCAAATAG
OsOryzain-specific	GACATGTCGATCGTGTCGTAC	CACAGTCAACTAGCTCCTGCTC

Supplementary Table 4. Criteria of *Laodelphax striatellus* resistance[9]

Injury level	Symptom
0	Unharmed
1	Very light harmed
3	The first and second leaves of most plants turn yellow
5	Plants turn noticeably yellow and dwarf, or more than half of the plants die
7	More than half of plants died, and the remaining plants were severely dwarf or near death
9	All plants die

Supplementary References

1. Richau KH*, et al.* Subclassification and biochemical analysis of plant papain-like cysteine proteases displays subfamily-specific characteristics. *Plant Physiol* **158**, 1583-1599 (2012).
2. Bozkurt T*, et al. Phytophthora infestans* effector AVRblb2 prevents secretion of a plant immune protease at the haustorial interface. *Proc Natl Acad Sci USA* **108**, 20832-20837 (2011).
3. Wang Y, Wang YC, Wang YM. Apoplastic proteases: powerful weapons against pathogen infection in plants. Plant communications 1, 100085 (2020).
4. Clark K*, et al.* An effector from the Huanglongbing-associated pathogen targets citrus proteases. *Nature Commun* **9**, 1718 (2018).
5. Lampl N, Alkan N, Davydov O, Fluhr R. Set-point control of RD21 protease activity by AtSerpin1 controls cell death in *Arabidopsis*. *Plant J* **74**, 498-510 (2013).
6. Ziemann S, *et al*. An apoplastic peptide activates salicylic acid signalling in maize. Nature plants **4**, 172-180 (2018).
7. Shangguan X*, et al.* A mucin-like protein of planthopper is required for feeding and induces immunity pesponse in plants. *Plant Physiol* **176**, 552-565 (2018).
8. Erb M, Meldau S, Howe GA. Role of phytohormones in insect-specific plant reactions. *Trends Plant Sci* **17**, 250-259 (2012).
9. Wu GR, Tao LY, Chen FY, Hu GW. On the screening methods of varietal resistance in rice to whitebacked planthopper, *Sogatella furcifera* Horvath. *Acta Entomological Sinica* **29**, 453-455 (1986).
