# Supplementary material for: Planthopper salivary sheath protein LsSP1 contributes to manipulation of rice plant defenses
Source: Nat Commun. 2023 Feb 10;14:737. doi: 10.1038/s41467-023-36403-5 (PMC9911632; doi:10.1038/s41467-023-36403-5)
Supplement: Supplementary file 7 — Reporting Summary [file 41467_2023_36403_MOESM7_ESM.pdf]

nature portfolio

Corresponding author(s): Chuan-Xi Zhang

Last updated by author(s): Jan 23, 2023

Reporting Summary

Nature Portfolio wishes to improve the reproducibility of the work that we publish. This form provides structure for consistency and transparency in reporting. For further information on Nature Portfolio policies, see our Editorial Policies and the Editorial Policy Checklist.

Statistics

For all statistical analyses, confirm that the following items are present in the figure legend, table legend, main text, or Methods section.

n/a	Confirmed	
☐	☒	The exact sample size (n) for each experimental group/condition, given as a discrete number and unit of measurement
☐	☒	A statement on whether measurements were taken from distinct samples or whether the same sample was measured repeatedly
☐	☒	The statistical test(s) used AND whether they are one- or two-sided<br>*Only common tests should be described solely by name; describe more complex techniques in the Methods section.*
☒	☐	A description of all covariates tested
☒	☐	A description of any assumptions or corrections, such as tests of normality and adjustment for multiple comparisons
☐	☒	A full description of the statistical parameters including central tendency (e.g. means) or other basic estimates (e.g. regression coefficient) AND variation (e.g. standard deviation) or associated estimates of uncertainty (e.g. confidence intervals)
☐	☒	For null hypothesis testing, the test statistic (e.g. F, t, r) with confidence intervals, effect sizes, degrees of freedom and P value noted<br>*Give P values as exact values whenever suitable.*
☒	☐	For Bayesian analysis, information on the choice of priors and Markov chain Monte Carlo settings
☒	☐	For hierarchical and complex designs, identification of the appropriate level for tests and full reporting of outcomes
☒	☐	Estimates of effect sizes (e.g. Cohen's d, Pearson's r), indicating how they were calculated

Our web collection on statistics for biologists contains articles on many of the points above.

Software and code

Policy information about availability of computer code

Data collection	Roche Light Cycler® 480 Real-Time PCR System for qPCR; GiGA-8d EPG amplifier for EPG; Leica confocal laser-scanning microscope SP8 for fluorescence microscopy; AI 680 image analyzer for WB visualization; SEM TM4000 II plus for scanning electron microscopy; Canon EOS 80D camera for photographing; LUMAZONE SOPHIA2048B cooled CDD imaging apparatus for LUC observation; BioTek microplate reader for fluorescence measurement.
Data analysis	The codes and softwares used in this study were commonly used. In detail, the SignalP 5.0 Server (https://services.healthtech.dtu.dk) was used to predict the presence of signal peptides and cleavage sites. Transmembrane domains were predicted by the TMHMM Server v. 2.0 (http://www. cbs.dtu.dk/services/TMHMM/). Protein conserved domains were predicted by InterPro 84.0-89.0 (http://www.ebi.ac.uk/interpro/). Best-matched homologs of planthopper species were aligned with the ClustalX program v1.81. Transcriptome data was mapped of reference genome sequences of O. sativa using HISAT v2.1.0 (https://daehwankimlab.github.io/hisat2/) with default parameters (-minintronlen 20). The low-quality alignments were filtered by SAMtools v1.7. Transcripts per million expression values were calculated using Cufflink v2.2.1. The DESeq2 v2.2.1(https://bioconductor.org/packages/release/bioc/vignettes/Glimma/inst/doc/DESeq2.html) was used for identify the differentially expressed genes with default parameters. DEGs were identified upon the thresholds of log2-ratio>1 and adjusted p-value<0.05. TBtools v1.0697 was used to perform KEGG analysis. PROBE 3.4 were used for analyze EPG data; The intensities of bands in immunoblot analyses were quantified using ImageJ software (version: 1.53e, https://imagej.nih.gov/). GraphPad Prism 9.0.0 software were used for graphing. R function plotPCA (github.com/franco-ye/TestRepository/blob/main/PCA_by_deseq2.R) was used to perform PCA analysis. DNAstar v8.0 was used to perform correlation analysis. SPSS Statistics 19 was applied to determine the statistical significance of survival distributions. MAFFT v7.450 was used to align PLCP sequences, and the gaps were further trimmed using Gblock v0.91b. RAxMLNG v0.9.0 was used to construct phylogenic tree.

For manuscripts utilizing custom algorithms or software that are central to the research but not yet described in published literature, software must be made available to editors and reviewers. We strongly encourage code deposition in a community repository (e.g. GitHub). See the Nature Portfolio guidelines for submitting code & software for further information.

Data

Policy information about availability of data

All manuscripts must include a data availability statement. This statement should provide the following information, where applicable:

- Accession codes, unique identifiers, or web links for publicly available datasets
- A description of any restrictions on data availability
- For clinical datasets or third party data, please ensure that the statement adheres to our policy

The sequencing data generated in this study have been deposited in the NCBI Sequence Read Archive under accession number PRJNA833487 [https://www.ncbi.nlm.nih.gov/bioproject/PRJNA833487] and PRJNA815455 [https://www.ncbi.nlm.nih.gov/bioproject/?term=PRJNA815455]. The TPM expression values of all genes generated from sequencing data can be found in Source data file. Sequence data can be found in GenBank under the following accession numbers: LsSP1, ON322955 [https://www.ncbi.nlm.nih.gov/nuccore/ON322955]; NlSP1, ASL05017 [https://www.ncbi.nlm.nih.gov/protein/ASL05017]; SfSP1, ON322954 [https://www.ncbi.nlm.nih.gov/nuccore/ON322954]; OsOryzain, NP_001389372.1 [https://www.ncbi.nlm.nih.gov/protein/NP_001389372]; LsMLP, ON568348 [https://www.ncbi.nlm.nih.gov/nuccore/ON568348]; NlMLP, KY348750 [https://www.ncbi.nlm.nih.gov/nuccore/KY348750]; SfMLP, AQP26312 [https://www.ncbi.nlm.nih.gov/protein/AQP26312]; hypothetical protein DAI22 06g016200, KAF2924946.1 [https://www.ncbi.nlm.nih.gov/protein/KAF2924946]; putative receptor-like protein kinase, XM_015785635 [https://www.ncbi.nlm.nih.gov/nuccore/XM_015785635]; SNF1-related protein kinase regulatory, XP_015639150.1 [https://www.ncbi.nlm.nih.gov/protein/XP_015639150.1]; polypyrimidine tract-binding protein, XP_015632933.1 [https://www.ncbi.nlm.nih.gov/protein/XP_015632933]; alpha-galactosidase, NP_001390973.1 [https://www.ncbi.nlm.nih.gov/protein/NP_001390973]; alpha-amylase, NP_001390734.1 [https://www.ncbi.nlm.nih.gov/protein/NP_001390734]; putative cysteine proteinase Os09g39160, BAD46641 [https://www.ncbi.nlm.nih.gov/protein/BAD46641.1]; zingipain-2 Os09g39090, XP_015611357 [https://www.ncbi.nlm.nih.gov/protein/XP_015611357]; putative cysteine proteinase Os09g39170, BAD46642 [https://www.ncbi.nlm.nih.gov/protein/BAD46642]; ervatamin-B Os09g39120, XP_015611254 [https://www.ncbi.nlm.nih.gov/protein/XP_015611254]; putative cysteine protease Os01g24570, BAD53944 [https://www.ncbi.nlm.nih.gov/protein/BAD53944]; and Os07g01800, BAC06931 [https://www.ncbi.nlm.nih.gov/protein/BAC06931]. The O. sativa reference genome was available in https://data.jgi.doe.gov/refine-downloadphytozome?)organism=Osativa&expanded=323. PLCP accessions were listed in Supplementary Fig. 8. The corresponding sequences of PLCPs were public available, and we also listed these sequences in Source data file,. Sequences of top 100 genes that abundantly expressed in L. striatellus salivary glands can be found in Source data file. Source data are provided with this paper.

Field-specific reporting

Please select the one below that is the best fit for your research. If you are not sure, read the appropriate sections before making your selection.

[x] Life sciences   [ ] Behavioural & social sciences   [ ] Ecological, evolutionary & environmental sciences

For a reference copy of the document with all sections, see nature.com/documents/nr-reporting-summary-flat.pdf

Life sciences study design

All studies must disclose on these points even when the disclosure is negative.

Sample size	No statistic method was used to pre-determine the sample size. Sample sizes were chosen based on established protocols and previous publications. Generally three independent biological replicates were done for each experiment. The insect bioassays (survival, fecundity, honeydew secretion, EPG recording analysis) were referred to Will et al. PNAS, 2008 (10.1073pnas.0703535104); Huang et al. J Proteome Res, 2016 (10.1021/acs.jproteome.6b00086); Ji et al., Plant Physiol, 2017 (10.1104/pp.16.01493); Guo et al. Current Biol, 2020 (10.1016/j.cub.2020.09.020).
Data exclusions	No data was excluded from the analyses, except for 1) offspring analysis: Some insects in CK, dsGFP-treatment, dsLsSP1-treatment have no offsprings. This phenomenon was commonly found in planthopper bioassay, possibly due to failure in fertilization. Therefore, we exclude the replicates without any offsprings in data analysis. 2) honeydew analysis: some insects were escaped from parafilm sachet during experiments, and these data were excluded from the analyses. All data excluded from the analyses were displayed in Source Data file, and labeled in red.
Replication	All attempts at replication were successful, and provided in the Source data file. Generally, experiments were repeated three times. For pull down assay, two independent replicates were performed.
Randomization	All samples were allocated randomly into experimental groups.
Blinding	All investigation were blinded to group allocation during data collection and analysis

Reporting for specific materials, systems and methods

We require information from authors about some types of materials, experimental systems and methods used in many studies. Here, indicate whether each material, system or method listed is relevant to your study. If you are not sure if a list item applies to your research, read the appropriate section before selecting a response.

Materials & experimental systems

n/a	Involved in the study
	☒ Antibodies
☒	Eukaryotic cell lines
☒	Palaeontology and archaeology
	☒ Animals and other organisms
☒	Human research participants
☒	Clinical data
☒	Dual use research of concern

Methods

n/a	Involved in the study
☒	ChIP-seq
☒	Flow cytometry
☒	MRI-based neuroimaging

Antibodies

Antibodies used

1. anti-OsOryzain serum (1:5000, WB; 1:200, IHC) was prepared using the synthetic peptides of VRMERNIKASSGKC and DVNRKNAKVVTIDSY, followed by injecting Rabbit via the custom service of Genscript, Nanjing, China.
2. anti-LsSP1 serum (1:5000, WB; 1:200, IHC) was prepared using the full amino acid ofLsSP1 (exclude signal peptide) fused with GST tag. The recombinant was expressed in Escherichia coli, followed by injecting Rabbit via the custom service of Huaan, Hangzhou, China.
3. 6x-His Tag Monoclonal Antibody (1:3000, WB), REF: MA1-21315; Vendor website: https://www.thermofisher.cn/cn/zh/antibody/product/6x-His-Tag-Antibody-clone-HIS-H8-Monoclonal/MA1-21315.
4. Horseradish peroxidase (HRP)-conjugated goat anti-rabbit IgG antibody (1:10,000, WB), REF: # 31460; Vendor website: https://www.thermofisher.com/antibody/product/Goat-anti-Rabbit-IgG-H-L-Secondary-Antibody-Polyclonal/31460
5. Horseradish peroxidase (HRP)-conjugated goat anti-mouse IgG antibody (1:10,000, WB), REF: # 31430; Vendor website: https://www.thermofisher.com/antibody/product/Goat-anti-Mouse-IgG-H-L-Secondary-Antibody-Polyclonal/31430

Validation

1. We validate the specificity of primary antibody by comparing the control sample (insect treated with dsGFP or WT plant) against the dsLsSP1-treated insect and OsOryzain KO mutant. The results were provided in Supplementary Figure 3 and Supplementary Figure 12.
2. The commercial antibodies were validated to be useful by manufacturer. Validation data / citation can be found on the manufacturers' website as follow: 1) HRP-conjugated goat anti-rabbit IgG antibody (https://www.thermofisher.cn/order/genome-database/dataSheetPdf?producttype=antibody&productsubtype=antibody_secondary&productId=31460&version=267); 2) HRP-conjugated goat anti-mouse IgG antibody (https://www.thermofisher.cn/order/genome-database/dataSheetPdf?producttype=antibody&productsubtype=antibody_secondary&productId=31430&version=267); 3) His-tag monoclonal antibody (https://www.thermofisher.cn/order/genome-database/dataSheetPdf?producttype=antibody&productsubtype=antibody_primary&productId=MA1-21315&version=267). The Antibody specificity of His-tag monoclonal antibody was demonstrated by manufacturers by detection of different targets fused to 6xHis tag in transiently transfected lysates tested. Relative detection of 6x-His tag was observed across different proteins fused with 6x-His in V5-H3-His (Lane 3) and Hisp65-YFP (Lane 4-6), using Anti-6x-His Tag Monoclonal Antibody (HIS.H8) (Product # MA1-21315) in Western Blot.

Animals and other organisms

Policy information about studies involving animals; ARRIVE guidelines recommended for reporting animal research

Laboratory animals

Laodelphax striatellus used in this study has been inbred following a sib-sib mating protocol for more than ten generations.
It is difficult to distinguish male and female at nymph stages. Therefore, we randomly select the male and female nymphs for analysis.
For investigating the influence of L. striatellus infestation on rice defense, the 4-5-leaf stage rice seedlings were infested by 5th instar L. striatellus nymphs (5 nymphs per plant). As it was difficult to distinguish insect sex at nymph stage, the 5th instar nymphs used in this study includes male and female individuals.
For tissue assay, carcasses (20), fat bodies (50), guts (50), and salivary glands (80) were dissected from the 5th instar nymphs. Similarly, testes (50) and ovaries (20) were collected from adult male and female L. striatellus, respectively. The number of insects in each sample was given in the parentheses above.
For survivorship assay, a group of 30-40 treated insects (3rd instar nymph) were treated with dsRNA and kept on 4-5 leaf stage rice seedlings.
For honeydew assay, 4th instar nymphs were treated with dsRNA. At 3-4 days post injection, the 4th instar nymphs get into the 5th instar stage, and the 5th instar nymphs were used for honeydew measurement.
For fecundity assay, the newly emerged male and female adults were treated with dsRNA. One day later, the insects were paired and allowed for oviposition for 10 days.
For salivary sheath analysis, 5th instar nymphs were allowed to fed on rice plants or artificial diets for 24 h.
For host choice test, the 4-5-leaf stage rice seedlings were infested by a group of 5 dsRNA-treated L. striatellus (5th instar nymph) for 24 h. Then, a group of 17 wild-type L. striatellus (4th instar nymph) were used for analysis.
For EPG recording, 4th instar nymphs were treated with dsRNA. At 3-4 days post injection, the 4th instar nymphs get into the 5th instar stage, and the 5th instar nymphs were used for analysis.
For evaluation of L. striatellus resistance, the 4-5 leaf stage seedlings were infested with 4th instar L. striatellus nymphs at ten insects per seedling.
For transcriptomic analysis, rice seedlings were infested by 5th instar L. striatellus, which pretreated with dsRNA at 4th instar stage.

Wild animals	This study did not involve wild animals.
Field-collected samples	This study did not involve samples collected from field.
Ethics oversight	We only use one insect species (Laodelphax striatellus) in this study. No ethical approval or guidance was required.

Note that full information on the approval of the study protocol must also be provided in the manuscript.
