# Supplementary material for: Planthopper salivary sheath protein LsSP1 contributes to manipulation of rice plant defenses
Source: Nat Commun. 2023 Feb 10;14:737. doi: 10.1038/s41467-023-36403-5 (PMC9911632; doi:10.1038/s41467-023-36403-5)
Supplement: Supplementary file 9 — Description of Additional Supplementary Files [file 41467_2023_36403_MOESM9_ESM.pdf]

File Name: Supplementary Data 1

Description: Analysis of top 100 abundant genes in salivary glands

File Name: Supplementary Data 2

Description: Differentially expressed genes between rice plants infested by ds*GFP*-treated and ds*LsSP1*-treated *Laodelphax striatellus*

File Name: Supplementary Data 3

Description: Differentially expressed genes in wild type Nipponbare plants after infestation by ds*LsSP1*-treated *Laodelphax striatellus*

File Name: Supplementary Data 4

Description: Differentially expressed genes in *oeSP1* plants after infestation by ds*LsSP1*-treated *Laodelphax striatellus*

File Name: Supplementary Data 5

Description: Differentially expressed genes in *koOry* plants after infestation by ds*LsSP1*-treated *Laodelphax striatellus*
